# Remodeling the gut-heart axis: Danggui Sini granule mitigates vasospastic coronary heart disease via microbiota-metabolite interactions

**DOI:** 10.3389/fcvm.2026.1833846

**Published:** 2026-05-22

**Authors:** Haipeng Tang, Zhiliang Sun, Shanshan Wang, Zhi Chen, Wenzhu Wang, Yang Wang, Jiyu Gong, Xiaoyan Xie, Wenyi Gao

**Affiliations:** 1 School of Pharmaceutical Sciences, Changchun University of Chinese Medicine, Changchun, China; 2 Jilin Ginseng Academy, Changchun University of Chinese Medicine, Changchun, China; 3 Department of Pharmacy, The Third Affiliated Hospital of Changchun University of Chinese Medicine, Changchun, China

**Keywords:** 12, 13-DHOME, coronary heart disease, Danggui Sini granule, gut microbiota, gut–heart axis, lipid metabolism, untargeted metabolomics

## Abstract

**Background:**

Coronary heart disease (CHD) remains a major global health burden, and residual cardiovascular risk persists despite guideline-based therapies. Danggui Sini Granule (DSG), a granule formulation derived from the classical Danggui Sini Decoction, has been widely used clinically to improve cardiovascular circulation and alleviate ischemic symptoms; however, its gut-heart-axis-related mechanisms remain insufficiently defined.

**Methods:**

A rat model of vasospastic CHD was established using a high-fat diet combined with acute environmental stress and pituitrin injection, followed by treatment with DSG at different doses. Pharmacodynamic effects were evaluated by electrocardiography, histopathology, serum lipids, and inflammatory markers. Gut microbial alterations were profiled using 16S rRNA sequencing. Serum metabolic changes were characterized by untargeted metabolomics. Network pharmacology and joint pathway enrichment were further used to cross-validate target prediction with metabolite-level readouts and to prioritize gut–microbiota–metabolism–phenotype links.

**Results:**

DSG attenuated ST-segment elevation, reduced myocardial histopathological injury, mitigated dyslipidemia, and lowered systemic inflammatory mediators, including TNF-α and IL-6. 16S rRNA analysis showed bidirectional microbiota modulation, with reduced opportunistic/pathogenic genera (e.g., *Escherichia*, *Staphylococcus*) and enrichment of potentially beneficial taxa (e.g., *Lactobacillus*, *Clostridium*). Metabolomics revealed partial mitigation of CHD-related disturbances, mainly in linoleic acid and arachidonic acid pathways, with restoration of levels of protective lipid mediators including 12,13-DHOME, 3-HIA, and PGE1. Joint pathway analysis integrating reversed metabolites with predicted targets consistently prioritized Lipid and atherosclerosis, PPAR-related metabolic regulation, PI3K-Akt/eNOS-associated vascular protection, and inflammation-related signaling (JAK-STAT and TNF/NF-κB). Furthermore, molecular docking suggested favorable binding affinities between the key components/metabolites (e.g., Quercetin, 12,13-DHOME, PGE1) and core targets within these pathways (including PIK3CA, NOS3, PPARG, TNF, and RELA).

**Conclusion:**

DSG demonstrated significant cardioprotective effects in vasospastic CHD rats, which were associated with coordinated gut microbiota remodeling, lipid-metabolic reprogramming, and modulation of inflammation-related pathways. These findings, further suggested by in silico molecular docking, provide a multidimensional framework for understanding the cardioprotective potential of DSG from a gut-heart-axis perspective. Given the associative nature of the current multi-omics data, key causal links should be validated in future targeted intervention studies.

## 1 Introduction

Coronary heart disease (CHD) remains a major global health challenge and a leading cause of morbidity and mortality worldwide (1). Pathophysiologically, CHD is primarily driven by coronary atherosclerosis, which is associated with lipid-metabolic dysregulation, oxidative stress, and chronic inflammation (2–4). Current guideline-based pharmacotherapy, including statins and antiplatelet agents, substantially reduces major adverse cardiovascular events (5). However, CHD also involves systemic cardiometabolic interactions across multiple organs. A substantial proportion of patients still show residual cardiovascular risk, partly related to persistent low-grade inflammation and metabolic dysregulation, while drug-related adverse effects may also limit long-term benefit in some populations (6, 7). Furthermore, emerging evidence on the gut-heart axis indicates that gut microbial dysbiosis can perturb host cardiometabolic homeostasis and is associated with atherosclerosis progression (8, 9). Recent advances have increasingly highlighted the profound impact of the gut microbiota and its circulating metabolites on the progression of cardiovascular diseases, establishing the gut-heart axis as a promising therapeutic target (10). Therefore, complementing standard care with interventions targeting microbiota-host metabolic interactions may provide additional therapeutic value in CHD management.

Danggui Sini Granule (DSG), a well-established classical prescription in Traditional Chinese Medicine (TCM), has been widely utilized in clinical practice to improve cardiovascular microcirculation and alleviate ischemic symptoms. Recent pharmacological studies confirm that DSG and its key active ingredients, such as ferulic acid, paeoniflorin, and cinnamaldehyde, can improve endothelial function, regulate thrombosis, and lessen ischemic damage (11–14). Because this herbal formula is taken orally, the gut and its local microbiota naturally serve as the first metabolic interface. Meanwhile, physiological studies show that severe environmental stress, like cold exposure, causes both cardiovascular spasms and gut flora imbalance. Therefore, we suspect that DSG protects the heart against stress-induced ischemia mainly by regulating the gut-heart axis and altering host metabolism (15).

Figuring out how complex herbal formulas work is always difficult. Today, systems biology and multi-omics tools help researchers solve this problem (16). For instance, untargeted metabolomics can quickly screen whole metabolic changes and identify pivotal lipid drivers in the progression of coronary artery disease (17, 18). Meanwhile, 16S rRNA sequencing is standard for observing gut bacteria, which helps reveal how medicinal herb-derived natural products undergo biotransformation to exert systemic cardioprotective effects (19). At the same time, network pharmacology helps predict how specific drug ingredients link to biological targets (20, 21). Even with these tools, we still do not know exactly how DSG affects the microbiota and metabolites during acute, stress-induced vasospastic CHD. This specific regulatory network remains unclear.

In this study, we aimed to test the therapeutic effects of DSG and explore its working mechanisms in rats with cold stress-induced vasospastic CHD. To mimic the real-world situation of patients who have basic metabolic problems and suffer from sudden cold stress, we designed a combined animal model. We first fed the rats a high-fat diet to cause dyslipidemia. Then, we used an ice-water bath to simulate sudden environmental cold and injected pituitrin to force acute coronary vasospasm. Finally, we combined 16S rRNA sequencing, serum metabolomics, and network pharmacology to track the multi-target mechanisms of DSG along the gut-heart axis. Additionally, in silico molecular docking was employed to explore the potential structural basis of these interactions. Through this approach, we hope to clarify the modern material basis of DSG and offer a new gut-metabolite viewpoint for treating stress-related heart diseases.

## 2 Materials and methods

### 2.1 Chemicals and reagents

To conduct our chemical analyses, we sourced the essential LC-MS solvents—specifically HPLC-grade acetonitrile, methanol, and isopropanol—directly from Fisher Scientific Corporation (Waltham, MA, USA). Other basic chemicals, including formic acid and dichloromethane, came from Aladdin Industrial Corporation (Shanghai, China), and we utilized Watsons purified water (Guangzhou, China) for all aqueous preparations. Regarding the animal modeling and pharmacological treatments, we procured Propranolol hydrochloride (Batch No. 241110) from Jiangsu Yunyang Group Pharmaceutical Co., Ltd. (Jiangsu, China) and Pituitrin (Batch No. S25398) from Shanghai Yuanye Bio-Technology Co., Ltd. (Shanghai, China). We ordered the required ELISA kits (Batch No. 202509) through Shanghai Youxuan Biological Technology Co., Ltd. (Shanghai, China). Additionally, Changchun Yisi Experimental Animal Co., Ltd. (Changchun, China) supplied the customized high-fat diet (Batch No. 20250716) for our rat cohorts. Finally, Hebei Shineway Pharmaceutical Co., Ltd. (Shijiazhuang, China) provided all the raw herbal medicines detailed in Table 1.

**Table 1:** The information on purchased herbal medicines.

No.	Latin name	English name	Chinese name	Batch No.
1	*Angelica sinensis (Oliv.) Diels*	Angelicae Sinensis Radix	Danggui	MX24031501
2	*Cinnamomum cassia Presl*	Cinnamomi Ramulus	Guizhi	PN2401012001
3	*Paeonia lactiflora Pall.*	Paeoniae Radix Alba	Baishao	BZ23111901
4	*Asarum heterotropoides Fr. Schmidt var. mandshuricum (Maxim.) Kitag.*	Asari Radix et Rhizoma	Xixin	TH24040304
5	*Glycyrrhiza uralensis Fisch.*	Glycyrrhizae Radix et Rhizoma	Gancao	HJQ23041501
6	*Akebia quinata (Thunb.) Decne.*	Akebiae Caulis	Mutong	HZ24040801
7	*Ziziphus jujuba Mill.*	Jujubae Fructus	Dazao	CX23120901

### 2.2 Formulation protocol of DSG

We formulated the DSG by strictly following a refined protocol previously established within our own research group (22). A mixture of seven medicinal herbs was prepared, consisting of 41.4 g of Angelica sinensis, 41.4 g of Cinnamomum cassia, 41.4 g of Paeonia lactiflora, 41.4 g of Asarum sieboldii, 27.6 g of Akebia quinata, 27.6 g of processed Glycyrrhiza uralensis, and 75 g of Ziziphus jujuba. The mixture was immersed in an appropriate volume of water and decocted to a final volume of 600 mL. The decoction was filtered through 200-mesh gauze and concentrated under reduced pressure to a relative density of 1.04–1.10 g/cm^3^. The concentrate was then spray-dried to obtain the DSG spray-dried powder. Subsequently, the powder was mixed with maltodextrin at a 1:1 ratio and processed into DSG using a dry granulation method. Doses were calculated based on the body surface area normalization method for a 70 kg adult (23). The granules were dissolved in warm water to prepare suspensions with concentrations of 0.62 g/mL, 1.24 g/mL, and 2.48 g/mL. The solutions were allowed to cool to room temperature before being administered via oral gavage.

### 2.3 Characterization of DSG by UPLC-MS

#### 2.3.1 Sample preparation

For the chemical profiling, we precisely weighed out 2.0 g of the finely ground DSG powder and thoroughly dispersed it into 50 mL of an aqueous methanol solution (methanol/water, 1:1, v/v). We then processed this suspension in an ultrasonic bath for 30 min. Following the sonication, we directly collected the resulting extract for the subsequent UHPLC-MS analysis.

#### 2.3.2 UHPLC-MS analysis of DSG

To separate the chemical constituents, we utilized a Waters ACQUITY™ UHPLC system (Waters Corporation, Manchester, UK) fitted with an ACQUITY BEH C18 column (50 mm  ×  2.1 mm, 1.7 μm). We formulated the mobile phase using purified water with 0.1% formic acid as solvent A, while acetonitrile served as solvent B. For each run, we injected a 5 μL sample and maintained a constant flow rate of 0.4 mL/min throughout the analysis. The gradient elution profile proceeded through the following stages: starting at 10% to 30% B for the first 5 min, ramping up to 80% B by 10 min, reaching 85% B at 12 min, and peaking at 95% B between 12 and 15 min. We held this 95% B concentration until 18 min before dropping it back to 10% B for the final equilibration up to 25 min.

Following chromatographic separation, the compounds directly entered a SYNAPT G2Si Q-TOF mass spectrometer (Waters Corp., Manchester, UK) operating with an electrospray ionization (ESI) source. We captured the mass data across a range of m/z 100 to 1,500 in both positive and negative ionization modes. To ensure optimal ionization, we configured the ESI source settings with a source temperature of 150 °C and a desolvation gas temperature of 500 °C. We applied a capillary voltage of +3.0 kV for positive mode (and −2.5 kV for negative mode), set the sampling cone voltage to 40 V, and maintained the nitrogen and cone gas flow rates at 900 L/h and 50 L/h, respectively.

Finally, to identify the detected substances, we matched our experimental MS and MS/MS spectra against established public databases. Specifically, we downloaded the MSMS_Public_EXP_NEG_VS17 and MSMS_Public_EXP_Pos_VS17 databases in MSP format from the Systems Omics Lab repository (https://systemsomicslab.github.io/compms/msdial/main.html#MSP) and processed the spectral matching via the MS-DIAL software platform (24).

### 2.4 Animals and treatments

To conduct the *in vivo* experiments, we sourced 36 healthy male Sprague-Dawley (SD) rats (body weight: 220–250 g) directly from Changchun Yisi Experimental Animal Co., Ltd. (Changchun, China). Following their arrival, we housed all subjects in a strictly regulated specific pathogen-free (SPF) facility. Rats were housed in six cages (six rats per cage) with corncob bedding in a climate-controlled facility (20–25 °C, 40%–55% relative humidity) under a 12 h light/dark cycle and were acclimatized for 7 days. Animals had *ad libitum* access to water and standard chow, except during model induction when a high-fat diet (HFD) was provided as described below. All procedures were conducted in accordance with national and institutional guidelines for animal care and use. All protocols involving animal subjects received prior ethical sanction from the Changchun University of Chinese Medicine Animal Ethics Committee (Approval No. 2025573, granted August 15, 2025). After acclimatization, rats were randomly allocated into six groups (*n* = 6 per group): Control, Model, Propranolol hydrochloride (Positive), DSG low-dose (DSG-L), DSG medium-dose (DSG-M), and DSG high-dose (DSG-H). All subsequent histopathological and biochemical evaluations were performed by investigators blinded to the group allocation.

The cold stress-exacerbated vasospastic CHD rat model was established with modifications based on a previous study (25). This tripartite model (HFD + cold stress + pituitrin) was deliberately chosen to closely replicate the multi-stage pathophysiology of clinical vasospastic CHD. Except for the Control group, rats were fed an HFD to induce a chronic baseline of dyslipidemia and endothelial dysfunction, which is the primary pathological foundation of CHD in clinical patients. Simultaneously, rats were subjected to daily cold stimulation in an ice-water bath (0–4 °C) for 5 min, which was designed to strictly mimic the acute environmental cold stress that frequently triggers vasospastic angina in clinical settings by prompting sympathetic overactivation and vascular reactivity (26). Following each 5 min cold exposure, animals were dried using warm air to minimize hypothermia and distress, and then allowed to recover at room temperature (20 °C). This daily procedure was maintained for a total of 6 weeks. During the last three days of week 6, Pituitrin (20 U/kg) was administered intraperitoneally once daily for three consecutive days to induce acute coronary artery spasms and temporary myocardial ischemia, thereby mimicking the acute anginal attacks seen in clinical scenarios (27). Together, this integrated model accurately reflects the clinical etiology of “chronic hyperlipidemia combined with acute cold-triggered vasospasm”, providing an optimal pathological environment to evaluate the efficacy of DSG.

From week 7, rats in the treatment groups received DSG granules or the positive drug by oral gavage once daily for 2 weeks, while the Control and Model groups received physiological saline. The dosages administered to the rats were calculated based on the clinical equivalent dose conversion formula between humans and animals (28). The specific dosages were as follows: Positive (Propranolol hydrochloride, 20 mg/kg), DSG-L (DSG, 8.9 g/kg), DSG-M (DSG, 17.8 g/kg), and DSG-H (DSG, 35.6 g/kg).

**Figure 1:**
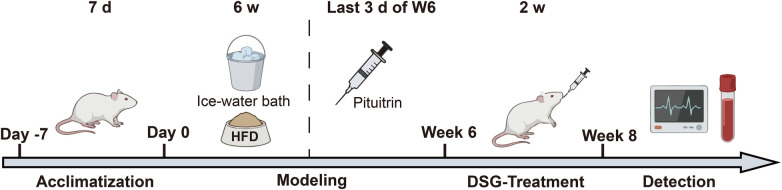
Schematic illustration of the experimental design. The timeline includes the induction of the cold stress-induced vasospastic CHD rat model (via HFD, ice-water bath, and pituitrin injection), followed by DSG administration and sample collection.

Animals were monitored daily for general condition (e.g., activity, posture, grooming, and food/water intake). Humane endpoints were predefined (e.g., >15%–20% body weight loss, persistent hypothermia, severe lethargy, dyspnea, or inability to access food/water), and animals meeting endpoints were euthanized immediately. After the treatment period, rats were anesthetized with pentobarbital sodium (40 mg/kg, i.p.) for electrocardiogram (ECG) examination and were euthanized by cervical dislocation under deep anesthesia; death was confirmed by cessation of heartbeat. Blood and tissue samples were immediately collected for biochemical and omics analyses. The anterior wall of the left ventricle was fixed in 4% paraformaldehyde solution for histopathological analysis. The experimental design, including modeling, administration, and grouping, is illustrated in Figure 1.

### 2.5 Electrocardiogram (ECG) detection

Rats were anesthetized with pentobarbital sodium (40 mg/kg, i.p.). After adequate anesthesia was confirmed (loss of pedal withdrawal reflex), rats were placed in the supine position and connected to a 16-channel physiological signal acquisition system for ECG recording. ST-segment changes were assessed.

### 2.6 Serum lipid analysis

Serum levels of total cholesterol (TC), high-density lipoprotein cholesterol (HDL-C), low-density lipoprotein cholesterol (LDL-C), and triglycerides (TG) were determined using an automated biochemical analyzer.

### 2.7 Hematoxylin and eosin (H&E) staining

Fresh heart tissue samples were preserved in 4% paraformaldehyde solution to maintain cellular structural integrity. After fixation, tissues underwent sequential dehydration to remove water, followed by wax infiltration prior to embedding. After embedding, sections were treated with hematoxylin solution to visualize nuclei, followed by eosin counterstaining to detect cytoplasm and extracellular matrix. The staining process was performed using an H&E staining kit (ServiceBio, Wuhan, China). Samples were then analyzed under a microscope, and images were captured.

### 2.8 Terminal deoxynucleotidyl transferase dUTP nick-end labeling (TUNEL) staining for cardiomyocyte apoptosis

Paraffin-embedded heart sections were deparaffinized with xylene and rehydrated through a graded ethanol series and distilled water. Sections were treated with Proteinase K, rinsed with PBS, and equilibrated at room temperature for 10 min. The TUNEL reaction mixture was added to the sections, followed by DAPI counterstaining for nuclei. Slides were mounted and observed under a fluorescence microscope for image acquisition.

### 2.9 Immunohistochemistry (IHC)

We processed the paraffin-embedded heart tissues by first boiling them in a citrate-based antigen retrieval buffer (pH 6.0, diluted at 1:1,000). After the slides cooled down, we applied the specific primary antibodies (at a 1:200 dilution) and left them to incubate in a 4 °C refrigerator overnight. The next morning, we brought the sections back to room temperature, washed them thoroughly, and added the HRP-conjugated secondary antibodies. To make the target proteins visible, we applied a DAB staining kit, which turns the positive areas brownish-yellow. We then used hematoxylin to counterstain the cell nuclei blue. Finally, we dehydrated, cleared, and mounted the slides before capturing images under a light microscope.

### 2.10 Enzyme-linked immunosorbent assay (ELISA) detection of Serum vasoactive factors and inflammatory cytokines

Blood samples were allowed to clot at room temperature for 30 min and then centrifuged at 3,000 × g for 15 min at 4 °C. The supernatant serum was collected for biochemical analysis. Levels of vasoactive factors—N-terminal pro-brain natriuretic peptide (NT-proBNP), Angiotensin II (Ang II), Endothelin-1 (ET-1), and Nitric Oxide (NO)—were measured using corresponding ELISA kits. Inflammatory cytokines, including Interleukin-6 (IL-6), Interleukin-1β (IL-1β), and Tumor Necrosis Factor-α (TNF-α), were also quantified. All procedures were strictly performed according to the manufacturer's instructions. Optical density (OD) values were measured using a microplate reader, and concentrations were calculated based on standard curves.

### 2.11 Network pharmacology

#### 2.11.1 Identification of bioactive ingredients and CHD-related targets

Using oral bioavailability (OB) ≥ 30% and drug-likeness (DL) ≥ 0.18 as screening criteria, the chemical constituents of Glycyrrhiza uralensis, Ziziphus jujuba, Angelica sinensis, Cinnamomum cassia, Asarum sieboldii, Akebia quinata, and Paeonia lactiflora in the DSG formula were retrieved from the TCMSP database (https://tcmspw.com/tcmsp.php) (29) as bioactive compounds. Targets were standardized using the UniProt database (https://www.uniprot.org/), and non-human targets were excluded to obtain the drug targets of DSG. Disease-related targets were collected from the OMIM database (https://mirror.omim.org/) and the GeneCards database (https://www.genecards.org/) using “Coronary Heart Disease” as the keyword, and duplicate entries were removed after merging.

#### 2.11.2 Construction of D-AI-T network and PPI network

The bioactive ingredients of DSG, their corresponding targets, and CHD-related genes were imported into Cytoscape 3.9.1 software to construct a “Drug-Active Ingredient-Target” (D-AI-T) network. DSG targets and disease targets were imported into Venny 2.1 (https://bioinfogp.cnb.csic.es/tools/venny) for intersection analysis to identify potential therapeutic targets of DSG for CHD. The overlapping targets were imported into the STRING database (https://string-db.org/cgi/input.pl) (30). A protein-protein interaction (PPI) network was constructed with “Homo sapiens” as the organism and a confidence score >0.4. The TSV file of the PPI network was downloaded and imported into Cytoscape 3.9.1. Subsequently, the MCODE plugin (Version 2.0.3) in Cytoscape was used for cluster analysis with the following parameters: Degree Cutoff = 2, Node Score Cutoff = 0.2, K-Core = 2, Max. Depth = 100. Modules with a Score ≥ 4.0 were selected as key functional modules for further analysis.

#### 2.11.3 Enrichment analysis

To investigate the signaling pathways and gene functions closely related to DSG treatment of CHD, common targets were analyzed using the Metascape platform (https://metascape.org/) (31) for Gene Ontology (GO) functional annotation and Kyoto Encyclopedia of Genes and Genomes (KEGG) pathway enrichment analysis. Visualization of GO and KEGG results was performed using the WeiShengXin platform (https://www.bioinformatics.com.cn/).

### 2.12 16S rRNA gene sequencing analysis

To profile the intestinal microbiome, we first isolated total genomic DNA directly from the collected rat feces relying on a FastPure Stool DNA Isolation Kit (MJYH, Shanghai, China). For the preliminary mechanistic exploration in this study, a sample size of *n* = 3 per group was utilized for 16S rRNA gene sequencing. Once we verified the quality of the extracted nucleic acids on a 1% agarose gel, we moved on to amplify the complete 16S rRNA sequence. For this PCR step, we employed the standard 27F (5′-AGRGTTYGATYMTGGCTCAG-3′) alongside the 1492R (5′-RGYTACCTTGTTACGACTT-3′) oligonucleotides, making sure to run three independent technical replicates per sample. Ultimately, we shipped these prepared samples to Majorbio Bio-Pharm Technology Co. Ltd. (Shanghai, China) to complete the downstream library construction and sequencing workflows. We set our ABI GeneAmp® 9,700 thermal cycler to start with a 3 min denaturation at 95 °C. This was followed by 27 amplification cycles (each consisting of 95 °C for 30 s, 60 °C for 30 s, and 72 °C for 30 s) and a final 10 min extension at 72 °C. After chilling the products to 4 °C, we ran them on a 2% agarose gel to excise and purify the target bands. We measured the DNA yield using a QuantiFluor™-ST fluorometer (Promega). The sequencing libraries were assembled with the SMRTbell Prep Kit 3.0 through standard damage repair, end repair, and adapter ligation steps. Finally, the libraries were sequenced on a PacBio Sequel IIe machine. We processed the raw paired-end data through the DADA2 pipeline (32) to remove noise, ultimately generating Amplicon Sequence Variants (ASVs) for our downstream diversity comparisons.

### 2.13 Serum metabolomics analysis

#### 2.13.1 Serum sample preparation

We processed the rat serum based on a reliable extraction method we established previously (33). In short, we pipetted 100 μL of each serum sample into a tube and vortexed it with 300 μL of ice–cold methanol to precipitate the proteins. We spun the mixtures in a 4 °C centrifuge at 15,000 × g for 15 min. We carefully took the supernatant, evaporated it completely under a gentle stream of nitrogen gas, and dissolved the remaining residue in 100 μL of chilled ultrapure water. After one last spin at 15,000 × g for another 15 min, we transferred 5 μL of the clear supernatant into vials for LC-MS injection.

#### 2.13.2 UHPLC-MS analysis

To acquire the comprehensive serum metabolic profiles, chromatographic separation was performed using an ACQUITY BEH C18 column (50 mm × 2.1 mm, 1.7 μm) maintained at 50 °C within the UHPLC-Q-TOF-MS framework. The binary solvent system comprised 0.1% aqueous formic acid (mobile phase A) and absolute acetonitrile (mobile phase B), flowing consistently at a rate of 0.4 mL/min. We programmed the gradient elution sequence as follows: starting with 3% B, the organic modifier was linearly increased to 70% over the initial 8 min and held steady until minute 10. Subsequently, solvent B climbed to 90% at 17 min and peaked at 100% by minute 18. After a 3-minute hold at this maximum organic composition (until 21 min), the system rapidly reverted to the initial 3% B within a single minute. A final 5-minute equilibration phase (total run time: 27 min) was applied to stabilize the column prior to the next injection.

Once separated, the molecules passed into a SYNAPT G2-Si Q-TOF mass spectrometer (Waters Corp., Manchester, UK) driven by an ESI source. We recorded full-scan data (m/z 100 to 1,500) and captured fragmentation spectra using a Fast DDA (Top 15) approach in resolution mode. Operating this electrospray interface required precise tuning across both polarities. Specifically, the capillary voltages were configured to 3.0 kV for positive ion detection and −2.5 kV for the negative mode. To ensure optimal ion transmission, the sampling cone was maintained at 40 V. Furthermore, thermal conditions within the source were tightly controlled, with the block temperature stabilized at 150 °C and the desolvation gas heated to 500 °C. Nitrogen and cone gas flows were kept steady at 900 L/h and 50 L/h, respectively. Finally, prior to sample runs, the instrument was calibrated with 0.5 mM sodium formate, while an infusion of leucine enkephalin (200 ng/mL) served as a continuous real-time lock-mass reference.

#### 2.13.3 Data processing and multivariate analysis

Raw data were preprocessed using Progenesis QI software (Nonlinear Dynamics, Newcastle, UK) for peak extraction, alignment, and normalization. A dataset containing sample ID, retention time (RT), m/z, and peak intensity was generated for subsequent statistical analysis. The dataset was imported into SIMCA 14.1 software to construct a Partial Least Squares Discriminant Analysis (PLS-DA) model. Variable Importance in Projection (VIP) values were calculated, and features with VIP > 1 were considered significant contributors to sample classification. Metabolites with a Fold Change (FC) > 1.5 and a Student's *t*-test *P* < 0.05 were screened as potential biomarkers associated with vasospastic CHD. Metabolite annotation was completed by searching the Human Metabolome Database (HMDB) using accurate m/z and MS/MS information. Pathway analysis was performed using the MetaboAnalyst 6.0 web tool (34).

### 2.14 Molecular docking analysis

To assess the interactions between the core regulators and the key targets identified in our joint analysis, molecular docking was performed. The structures of the key active ingredients (Quercetin and Kaempferol, selected as primary topological hubs based on degree centrality evaluations) were obtained from the TCMSP database, while the structures of the crucial endogenous metabolites 12,13-dihydroxy-9Z-octadecenoic acid (12,13-DHOME) and prostaglandin E1 (PGE1) were retrieved from the PubChem database (35). The high-resolution x-ray crystal structures of the core target proteins, including PIK3CA, NOS3, PPARG, TNF, and RELA, were downloaded from the RCSB Protein Data Bank (PDB). Prior to docking, the target protein structures were preprocessed using PyMOL software to remove water molecules and original co-crystallized ligands. AutoDockTools 1.5.6 was utilized to add polar hydrogens and compute Gasteiger charges for both proteins and ligands, converting them into the PDBQT format. The molecular docking simulations were executed using AutoDock Vina (36). A calculated binding free energy ≤−5.0 kcal/mol was considered the threshold indicating favorable binding affinity between the ligand and the receptor. The optimal docking conformations with the lowest binding energies were ultimately visualized and analyzed using PyMOL software.

### 2.15 Statistical analysis

We used GraphPad Prism 9 (GraphPad Software, San Diego, CA, USA) for all statistical graphing and calculations. All continuous variables are expressed as the mean ± standard deviation (SD). To compare differences among multiple groups, we applied a one-way analysis of variance (ANOVA), and then used Tukey's test for *post hoc* comparisons. We set the statistical significance threshold at a two-sided *p*-value of less than 0.05. For mechanistic exploration at the pathway level, we ran joint pathway analyses via the MetaboAnalyst 6.0 web platform (34). Additionally, we calculated Spearman's rank correlation coefficients to evaluate the links between the gut flora, serum metabolites, and disease phenotypes. In this network, relationships meeting the criteria of |*ρ*| > 0.6 and *P* < 0.05 were visualized as heatmaps with the help of OmicShare tools, a free interactive online platform for data analysis (https://www.omicshare.com/tools) (37).

## 3 Results

### 3.1 Chemical characterization of DSG

**Figure 2:**
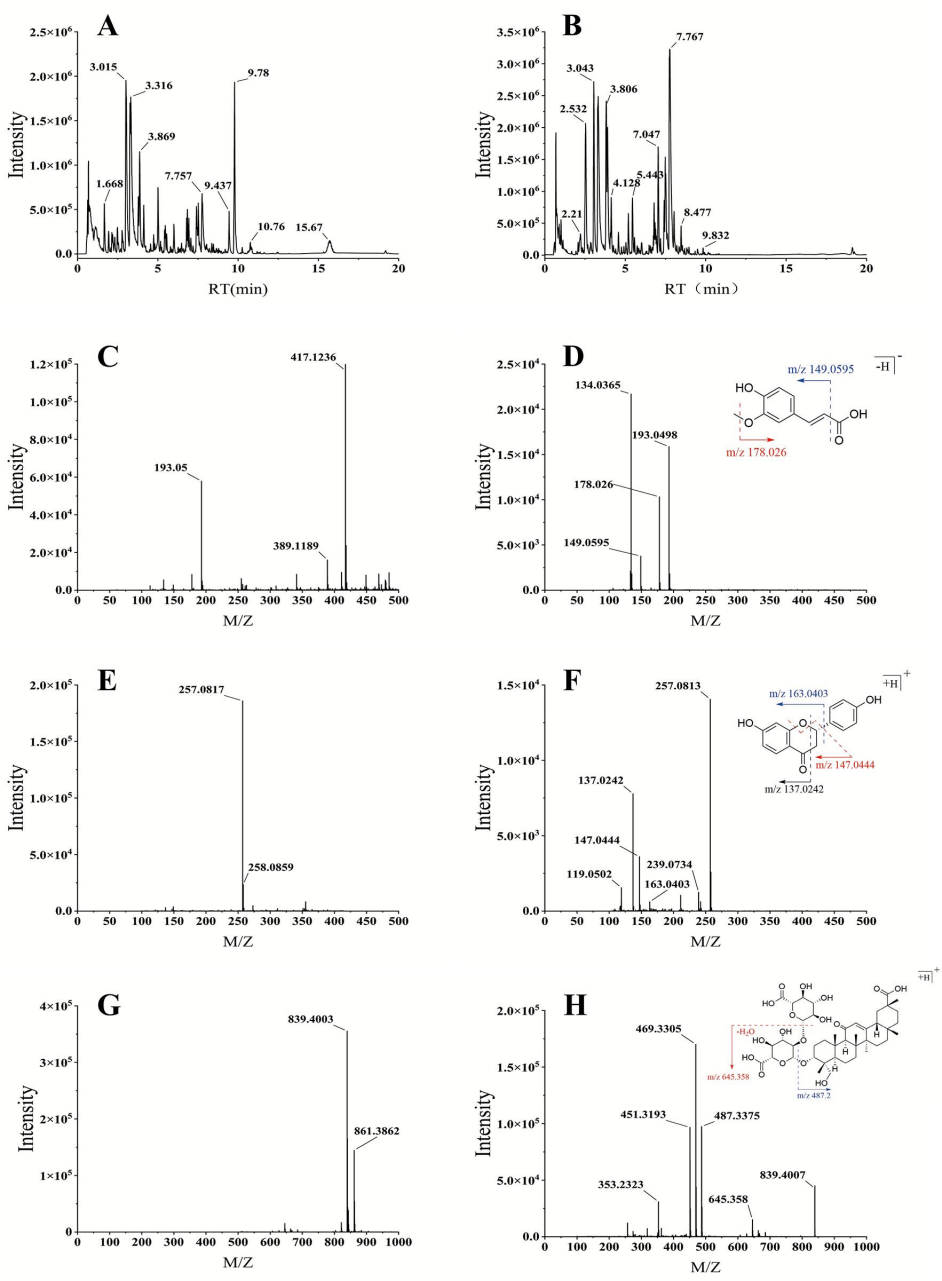
Chemical characterization of DSG by UPLC-Q-TOF-MS. **(A,B)** Base peak chromatograms (BPC) of DSG in positive **(A)** and negative **(B)** ionization modes. **(C–H)** MS and MS/MS spectra, alongside proposed fragmentation pathways, of representative compounds: Ferulic acid **(C,D)**, Liquiritigenin **(E,F)**, and Licorice Saponin G2 **(G,H)**.

We ran the DSG extract through a UHPLC-Q-TOF-MS system under both positive and negative ion modes to map its chemical profile. The resulting base peak chromatograms (BPC) are provided in Figures 2A,B. By matching our MS data with reference databases, we successfully annotated 105 different constituents (detailed in Table 2). The majority of these detected substances belong to phenolic acids, flavonoids, and triterpenoid saponins. Below, we break down the identification steps for a few representative compounds to demonstrate our annotation strategy.

**Table 2:** The chemical composition of DSG detected by UHPLC-MS.

No.	Compound name	Molecular Formula	Adducts	RT (min)	Theoretical m/z	Detected m/z	Mass error (ppm)
1	Glycolic acid	C2H4O3	[M − H]−	0.71	75.0088	75.0087	−1.33
2	D-(-)-Quinic acid	C7H12O6	[M − H]−	0.76	191.0561	191.0562	0.52
3	Lipoic acid reduced	C8H16O2S2	[M − H]−	0.86	207.0519	207.0523	1.93
4	Picolinic acid	C6H5NO2	[M + H]+	0.88	124.0393	124.0392	−0.81
5	Epicatechin-3-o-gallate	C22H18O10	[M − H]−	0.93	441.0827	441.0788	−8.84
6	(-)Catechin	C15H14O6	[M + H]+	0.93	291.0863	291.0879	5.50
7	Aconitic Acid	C6H6O6	[M + NH4]+	0.96	192.0503	192.0497	−3.12
8	Olivetoric acid	C26H32O8	[M + NH4]+	1.00	490.2435	490.2427	−1.63
9	Adenosine	C10H13N5O4	[M + H]+	1.00	268.1040	268.1054	5.22
10	Lobaric acid	C25H28O8	[M + NH4]+	1.01	474.2122	474.2139	3.58
11	Salsoline	C11H15NO2	[M + H]+	1.04	194.1176	194.1183	3.61
12	5-(1,2-dithiolan-3-yl)pentanoic acid	C8H14O2S2	[M + Na]+	1.21	229.0327	229.0335	3.49
13	trans-Cinnamic acid	C9H8O2	[M + H]+	1.24	149.0602	149.0612	6.71
14	Gallic acid hexoside	C13H16O10	[M − H]−	1.28	331.0671	331.0671	0.00
15	Epicatechin gallate	C22H18O10	[M + NH4]+	1.34	460.1238	460.1263	5.43
16	Vanillic acid	C8H8O4	[M − H]−	1.36	167.0350	167.0349	−0.60
17	Notatic acid	C18H16O7	[M + NH4]+	1.44	362.1234	362.1244	2.76
18	Benzoic acid hexoside	C13H16O9	[M − H]−	1.46	315.0722	315.0692	−9.52
19	Higenamine	C16H17NO3	[M + H]+	1.52	272.1281	272.1306	9.19
20	DL-4-Hydroxy-3-methoxymandelic acid	C9H10O5	[M − H]−	1.54	197.0455	197.0453	−1.01
21	Apigenin-diglucuronide	C27H26O17	[M − H]−	1.55	621.1097	621.1074	−3.70
22	3-Formylindole	C9H7NO	[M + H]+	1.68	146.0600	146.0597	−2.05
23	Tryptophan	C11H12N2O2	[M − H]−	1.68	203.0826	203.0808	−8.86
24	Indole	C8H7N	[M + H]+	1.68	118.0651	118.0644	−5.93
25	D-Tryptophan	C11H12N2O2	[M + H]+	1.68	205.0972	205.0962	−4.88
26	Homovanillic acid	C9H10O4	[M − H]−	1.69	181.0506	181.0510	2.21
27	Propyl gallate	C10H12O5	[M − H]−	1.77	211.0612	211.0624	5.69
28	Dihydroluteolin	C15H12O6	[M + H]+	1.84	289.0707	289.0729	7.61
29	Gallic acid	C7H6O5	[M − H]−	2.08	169.0142	169.0138	−2.37
30	Cinnamic acid	C9H8O2	[M − H2O − H]−	2.16	147.0452	147.0456	2.72
31	Asperulosidic acid	C18H24O12	[M + H]+	2.34	433.1341	433.1334	−1.62
32	Boldine	C19H21NO4	[M + H]+	2.41	328.1543	328.1517	−7.92
33	5-Caffeoylquinic acid	C16H18O9	[M + H]+	2.47	355.1000	355.0973	−7.60
34	3-Methylbenzoic acid	C8H8O2	[M − H]−	2.65	135.0452	135.0454	1.48
35	Caffeic acid	C9H8O4	[M − H]−	2.68	179.0350	179.0350	0.00
36	Apigenin-6-C-glucoside	C27H30O15	[M − H]−	2.69	593.1512	593.1501	−1.85
37	3,4-Dihydroxyphenylacetic acid	C8H8O4	[M − H]−	2.92	167.0350	167.0349	−0.60
38	Divaric acid	C10H12O4	[M + H]+	3.04	197.0808	197.0795	−6.60
39	Albiflorin	C23H28O11	[M + H]+	3.04	481.1704	481.1698	−1.25
40	Apiin	C26H28O14	[M − H]−	3.14	563.1406	563.1386	−3.55
41	Isoschaftoside	C26H28O14	[M + H]+	3.15	565.1552	565.1498	−9.55
42	3-O-Caffeoylquinic acid methyl ester	C17H20O9	[M + Na]+	3.24	391.1000	391.1027	6.90
43	4-hydroxybenzaldehyde	C7H6O2	[M − H]−	3.29	121.0295	121.0299	3.30
44	Mellein	C10H10O3	[M + H]+	3.30	179.0703	179.0695	−4.47
45	6,4′-Dihydroxyflavone	C15H10O4	[M + H]+	3.49	255.0652	255.0649	−1.18
46	Neohesperidin Dihydrochalcone	C28H36O15	[M + Na]+	3.56	635.1946	635.1951	0.79
47	p-Coumaric acid	C9H8O3	[M − H]−	3.58	163.0401	163.0400	−0.61
48	(Z)-2-octylpent-2-enedioic acid	C13H22O4	[M − H]−	3.59	241.1445	241.1444	−0.41
49	Liquiritigenin	C15H12O4	[M + H]+	3.87	257.0808	257.0817	3.50
50	Palmitic acid	C16H32O2	[M + H]+	3.88	257.2500	257.2520	7.77
51	Liquiritin	C21H22O9	[M + Na]+	3.90	441.1156	441.1199	9.75
52	Ferulic acid	C10H10O4	[M − H]−	4.02	193.0506	193.0500	−3.11
53	Hydroxysebacic acid	C10H18O5	[M − H]−	4.07	217.1081	217.1076	−2.30
54	5-Methoxy-3-indoleacetic acid	C11H11NO3	[M − H]−	4.17	204.0666	204.0654	−5.88
55	Luteolin 7-apiosyl-(1->2)-glucoside	C26H28O15	[M + Na]+	4.49	603.1320	603.1299	−3.48
56	9-(2,3-dihydroxypropoxy)-9-oxononanoic acid	C12H22O6	[M + Na]+	4.50	285.1309	285.1302	−2.46
57	Naringenin	C15H12O5	[M + H]+	4.75	273.0757	273.0748	−3.30
58	Emodin	C15H10O5	[M + H]+	4.80	271.0601	271.0586	−5.53
59	Coniferaldehyde	C10H10O3	[M + H]+	5.03	179.0703	179.0695	−4.47
60	Kaempferol-7-O-hexoside	C21H20O11	[M − H]−	5.35	447.0933	447.0890	−9.62
61	p-Methoxycinnamic acid ethyl ester	C12H14O3	[M + H]+	5.49	207.1016	207.1018	0.97
62	Coumarin	C9H6O2	[M + H]+	5.53	147.0441	147.0427	−9.52
63	Formononetin-7-O-glucoside	C22H22O9	[M + HCOO]−	5.57	475.1246	475.1283	7.79
64	Ononin	C22H22O9	[M + H]+	5.58	431.1337	431.1368	7.19
65	3-(4-hydroxy-5-oxo-3-phenyl-2H-furan-2-yl)propanoic acid	C13H12O5	[M − H]−	5.61	247.0612	247.0607	−2.02
66	Kaempferol	C15H10O6	[M − H]−	5.87	285.0405	285.0426	7.37
67	Benzylsuccinic acid	C11H12O4	[M − H]−	6.02	207.0663	207.0649	−6.76
68	3,5-Di-caffeoylquinic acid	C25H24O12	[M + Na]+	6.38	539.1160	539.1161	0.19
69	Quercetin	C15H10O7	[M − H]−	6.44	301.0354	301.0340	−4.65
70	2'-O-methylisoliquiritigenin	C16H14O4	[M + H]+	6.93	271.0965	271.0945	−7.38
71	Alisol F	C30H48O5	[M + Na]+	7.08	511.3394	511.3441	9.19
72	Juarezic Acid	C11H10O2	[M − H]−	7.24	173.0608	173.0605	−1.73
73	Tectorigenin	C16H12O6	[M + H]+	7.33	301.0707	301.0711	1.33
74	Licoricesaponin G2	C42H62O17	[M + H]+	7.52	839.4060	839.4003	−6.79
75	Linoleic acid	C18H32O2	[M + H]+	7.55	281.2500	281.2512	4.27
76	7-O-Methylchrysin	C16H12O4	[M + H]+	7.67	269.0808	269.0784	−8.92
77	DL-5-Hydroxylysine	C6H14N2O3	[M − H]−	7.69	161.0932	161.0939	4.35
78	Licoricesaponin H2	C42H62O16	[M + H]+	7.78	823.4111	823.4136	3.04
79	Glycyrrhizic acid	C42H62O16	[M + Na]+	7.88	845.3930	845.3937	0.83
80	6-Ethoxy-3 (4'-hydroxyphenyl)-4-methylcoumarin	C18H16O4	[M − H]–	7.94	295.0976	295.0998	7.46
81	Quercetin 3-O-beta-D-xylopyranoside	C20H18O11	[M + Na]+	8.06	457.0700	457.0692	−1.75
82	2,2'-(Tetradecylimino)diethanol	C18H39NO2	[M + H]+	8.09	302.3054	302.3071	5.62
83	FA 18:2 + 2O	C18H32O4	[M − H]−	8.27	311.2228	311.2203	−8.03
84	Xanthoxylin	C10H12O4	[M + H]+	8.37	197.0808	197.0795	−6.60
85	7,8-Dihydroxy-4-methylcoumarin	C10H8O4	[M + H]+	8.42	193.0495	193.0493	−1.04
86	Methyl trans-cinnamic acid	C10H10O2	[M + H]+	8.66	163.0754	163.0743	−6.75
87	Vanillin	C8H8O3	[M + H]+	8.80	153.0546	153.0532	−9.15
88	8-Prenylnaringenin	C20H20O5	[M + H]+	8.80	341.1383	341.1367	−4.69
89	7-Hydroxy-4-methylcoumarin	C10H8O3	[M + H − H2O]+	8.85	177.1600	177.1584	−9.03
90	Licoflavone C	C20H18O5	[M + H]+	9.04	339.1227	339.1239	3.54
91	Deacetylgedunin	C26H32O6	[M − H]−	9.07	439.2126	439.2095	−7.06
92	1-(4-Methoxyphenyl)-1-pentene-3-one	C12H14O2	[M + H]+	9.21	191.1067	191.1071	2.09
93	6,7-dimethoxy-2H-chromen-2-one	C11H10O4	[M + H]+	9.35	207.0652	207.0641	−5.31
94	Phosphocholine	C5H15ClNO4P	[M + H]+	9.41	184.0740	184.0756	8.69
95	gamma-Glutamyltyrosine	C14H18N2O6	[M + H]+	9.45	311.1236	311.1224	−3.86
96	Quercetin-pentoside	C27H28O16	[M − H]−	9.80	607.1305	607.1343	6.26
97	Licoricidin	C26H32O5	[M + H]+	9.94	425.2323	425.2288	−8.23
98	LysoPhosphatidylcholine 16:0	C24H50NO7P	[M + H]+	10.16	496.3398	496.3416	3.63
99	Phosphatidylethanolamine lyso 18:0	C23H48NO7P	[M − H]−	10.17	480.3096	480.3118	4.58
100	Pygenic acid A	C30H48O4	[M − H]−	10.29	471.3480	471.3467	−2.76
101	18β-Glycyrrhetinic acid	C30H46O4	[M + H]+	10.34	471.3469	471.3472	0.64
102	ent-11beta-Hydroxyatis-16-ene-3,14-dione	C20H28O3	[M + Na]+	10.36	339.1900	339.1882	−5.31
103	Corynoxeine	C22H26N2O4	[M + H]+	11.04	383.1965	383.2000	9.13
104	Kaempferol-7-O-neohesperidoside	C27H30O15	[M + H]+	11.06	595.5000	595.4990	−1.68
105	Deoxycholic acid	C24H40O4	[M + H]+	15.35	393.2999	393.2987	−3.05

First, let's look at Compound #52. It showed a retention time (RT) of 4.02 min and produced a strong [M − H]⁻ signal at m/z 193.0500. As seen in Figure 2C, this peak co-eluted alongside other ions at m/z 389.1189 and 417.1236. When we examined the MS/MS fragmentation of the 193.0500 precursor (Figure 2D), the base peak appeared at m/z 193.0498. We also captured two distinct secondary fragments: one at m/z 149.0595 caused by decarboxylation, and another at m/z 178.0260 resulting from a missing methyl group. Because this specific cleavage behavior perfectly matches the known mass spectrometry profile of ferulic acid, we confidently marked Compound #52 as this specific phenolic acid.

Another example is Compound #49 (RT = 3.87 min), whose mass spectra are displayed in Figures 2E,F. During the full MS1 scan, it yielded a very clear [M + H]⁺ molecular ion at m/z 257.0817. We verified the reliability of this signal by checking its carbon-13 isotope peak at m/z 258.0859, which aligned well with theoretical predictions. Moving to the fragmentation stage, the precursor ion lost a water molecule to create a fragment at m/z 239.0734, while the detachment of the B-ring generated an ion at m/z 163.0403. Further C-ring cleavage events produced m/z 147.0444 (loss of the B-ring plus a single-bonded oxygen) and m/z 137.0242 (which retains the A-ring and a fragment of the C-ring). All these structural clues pointed directly to the flavonoid liquiritigenin.

As shown in Figure 2G, the mass spectrum of Compound #74 at RT 7.52 min exhibited ions at *m/z* 839.4003 and 861.3862, corresponding to the [M + H]⁺ and [M + Na]⁺ ions, respectively. Figure 2H displays the MS/MS spectrum of this compound. The fragment at *m/z* 487.3375 originated from the loss of two glucuronic acid moieties [M − 2GlcA + H]⁺ from the precursor. Subsequent losses of one and two water molecules from this fragment yielded ions at *m/z* 469.3305 and *m/z* 451.3193, respectively. Additionally, the ion at *m/z* 645.3580 corresponded to the fragment formed by the loss of one glucuronic acid and one water molecule from the precursor [M − GlcA − H₂O + H]⁺. Accordingly, this compound was identified as the triterpenoid saponin Licorice Saponin G2.

### 3.2 Effects of DSG on coronary heart disease

#### 3.2.1 Effect of DSG on body weight, cardiac index, and serum lipid profiles

**Figure 3:**
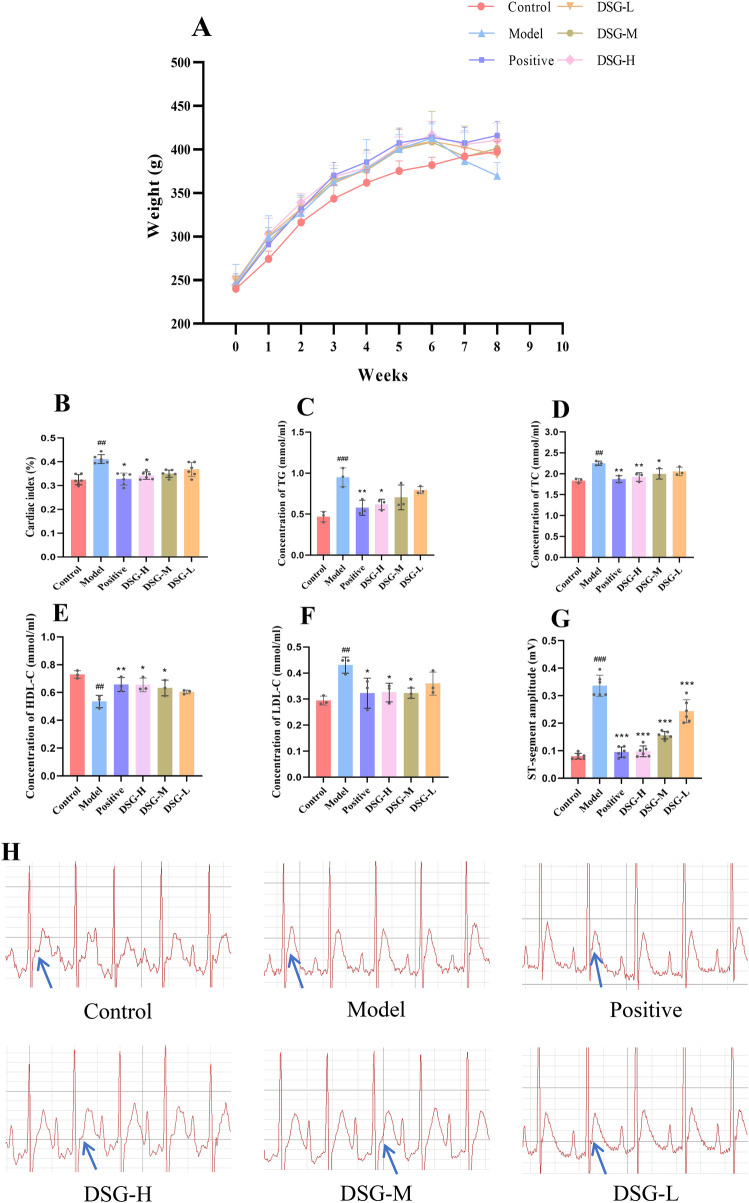
Effects of DSG on body weight, cardiac index, serum lipids, and electrocardiogram (ECG) in CHD rats. **(A)** Body weight. **(B)** Cardiac index. **(C–F)** Serum levels of TG, TC, HDL-C, and LDL-C. **(G)** Quantitative analysis of ST-segment elevation. **(H)** Representative ECG recordings (blue arrows indicate the ST-segment). Data are presented as mean ± SD (*n* = 3–6 per group). The exact sample size for each specific group and parameter is represented by the individual data points displayed in the scatter bar plots to illustrate the data distribution. # *P* < 0.05, ## *P* < 0.01, ### *P* < 0.001 vs. the Control group; * *P* < 0.05, ** *P* < 0.01, *** *P* < 0.001 vs. the Model group.

As shown in Figure 3A, compared to the Control group, the Model and treatment groups exhibited a decreasing trend in body weight following the 6-week modeling period. However, after drug administration, the DSG-H, DSG-M, and Positive groups showed a trend of weight recovery compared to the Model group. As illustrated in Figures 3B–F, the cardiac index and serum levels of TG, TC, and LDL-C were significantly elevated in the Model group compared to the Control group (all *P* < 0.01). In contrast, the level of HDL-C was significantly reduced in the Model group (*P* = 0.0014). Following intervention, DSG-H significantly attenuated the increases in cardiac index (*P* = 0.0164), TG (*P* = 0.0107), TC (*P* = 0.0068), and LDL-C (*P* = 0.0407), while effectively restoring HDL-C levels (*P* = 0.0453).

#### 3.2.2 DSG ameliorates ECG abnormalities

As shown in Figures 3G,H, the ST-segment of the ECG in the Control group was close to the isoelectric line. In contrast, the Model group exhibited significant ST-segment elevation compared to the Control group (*P* < 0.001). Notably, DSG intervention significantly reduced the ST-segment elevation compared to the Model group, with the most pronounced improvement observed in the DSG-H group (*P* < 0.001). Statistical analysis revealed a significant difference between the DSG-H group and the Model group, suggesting that DSG effectively ameliorates ECG abnormalities.

#### 3.2.3 DSG reduces cardiomyocyte apoptosis and ameliorates histopathological damage

**Figure 4:**
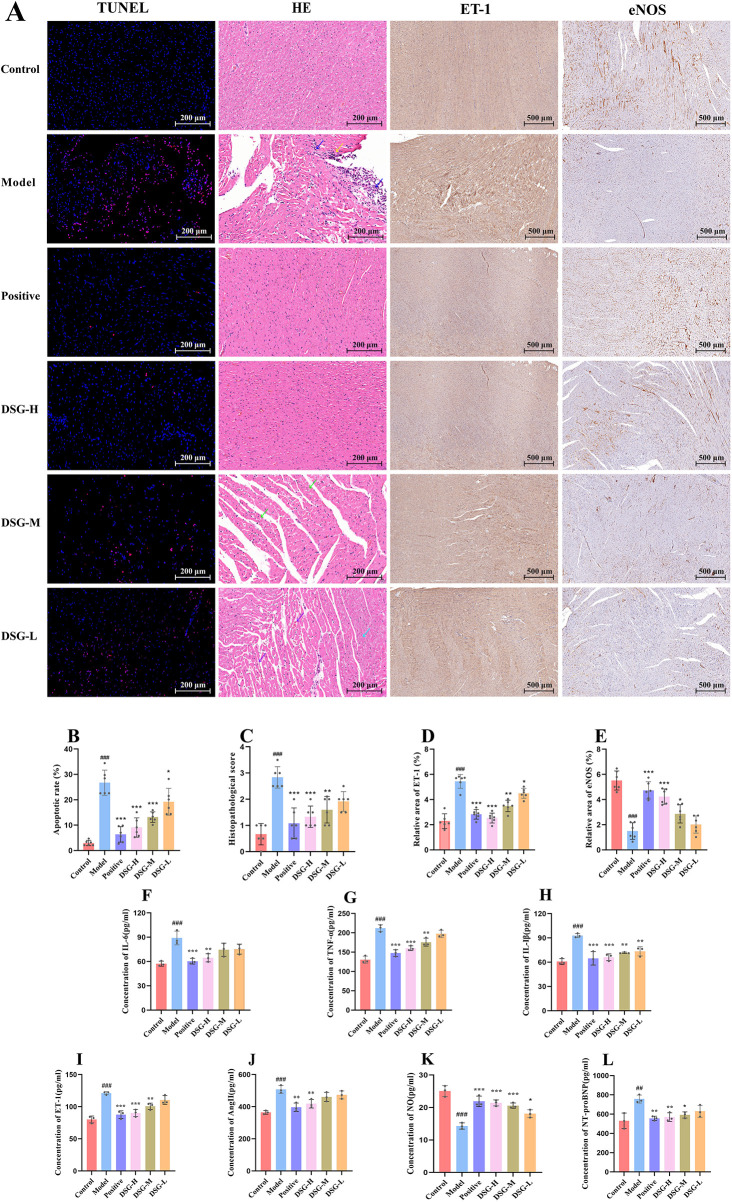
DSG attenuates myocardial injury and apoptosis and regulates vasoactive/inflammatory factors. **(A)** Representative images of TUNEL, H&E, and IHC (ET-1 and eNOS) staining. In TUNEL staining, red fluorescence indicates apoptotic nuclei (TUNEL-positive). In H&E images, blue and yellow arrows indicate focal lymphocyte infiltration and cardiomyocyte necrosis, respectively. Brownish-yellow staining indicates positive immunoreactivity for ET-1/eNOS. **(B)** Quantification of apoptotic index (%) based on TUNEL staining. **(C)** Quantification of histopathological score based on H&E staining. **(D,E)** Quantification of the relative positive area (%) of ET-1 and eNOS in IHC staining. **(F–L)** Serum levels of inflammatory cytokines (IL-6, IL-1β, TNF-α) and vasoactive factors (NT-proBNP, NO, Ang II, ET-1). Data are presented as mean ± SD (*n* = 3–6 per group). The exact sample size for each specific group and parameter is represented by the individual data points displayed in the scatter bar plots to illustrate the data distribution. Scale bars: 200 μm for TUNEL and H&E; 500 μm for IHC (ET-1 and eNOS). # *P* < 0.05, ## *P* < 0.01, ### *P* < 0.001 vs. the Control group; * *P* < 0.05, ** *P* < 0.01, *** *P* < 0.001 vs. the Model group.

To evaluate the cardioprotective effect of DSG, TUNEL assay and H&E staining were performed. As shown in Figures 4A,B, the Model group displayed a significant increase in TUNEL-positive cells compared with the Control group (*P* < 0.001), indicating enhanced cardiomyocyte apoptosis. DSG treatment significantly reduced the apoptotic index, with the most pronounced effect observed in the DSG-H group compared to the Model group (*P* < 0.001).

Consistent with the apoptosis data, H&E staining (Figures 4A,C) showed marked myocardial injury in the Model group (*P* < 0.001), including focal lymphocyte infiltration (blue arrows) and cardiomyocyte necrosis (yellow arrows). DSG administration significantly alleviated these pathological changes, with the strongest improvement observed in the DSG-H group (*P* < 0.001). These findings indicate that DSG attenuates myocardial injury in the CHD rat model.

#### 3.2.4 DSG modulates ET-1 and eNOS protein levels in heart tissue

IHC staining was used to assess ET-1 and eNOS expression in cardiac tissue, with positive signals indicated by brownish-yellow staining. As shown in Figures 4A,D, ET-1 expression was significantly increased in the Model group compared with the Control group (*P* < 0.001). In contrast, eNOS expression was significantly reduced in the Model group (*P* < 0.001) (Figures 4A,E).

Compared with the Model group, DSG treatment significantly reduced ET-1-positive area across all dose groups (all *P* < 0.05) and significantly increased eNOS-positive area in the DSG-M (*P* = 0.0195) and DSG-H groups (*P* < 0.001), while the DSG-L group showed an increasing trend in eNOS expression without statistical significance (*P* = 0.7760). The positive-control group also significantly decreased ET-1 (*P* < 0.001) and restored eNOS expression (*P* < 0.001). These results suggest that DSG may improve endothelial-related vasoactive factor balance in CHD rats.

#### 3.2.5 DSG mitigates serum inflammatory response and regulates vasoactive factors

We next measured serum levels of inflammatory cytokines (IL-6, IL-1β, and TNF-α) and vasoactive factors (NT-proBNP, NO, Ang II, and ET-1) to confirm the cardiovascular benefits of DSG. Looking at Figures 4F–L, the Model group showed a sharp increase in both pro-inflammatory cytokines and vasoconstrictive factors (ET-1 and Ang II), alongside a severe drop in NO levels (all *P* < 0.001). Treating the animals with DSG successfully reversed these trends. Compared with the Model group, the DSG-H group maintained significantly lower levels of inflammatory and vasoconstrictive markers and restored NO levels (all *P* < 0.01). This protective effect followed a dose-dependent pattern, with the DSG-H group demonstrating the most significant therapeutic efficacy.

### 3.3 Network pharmacology analysis

**Figure 5:**
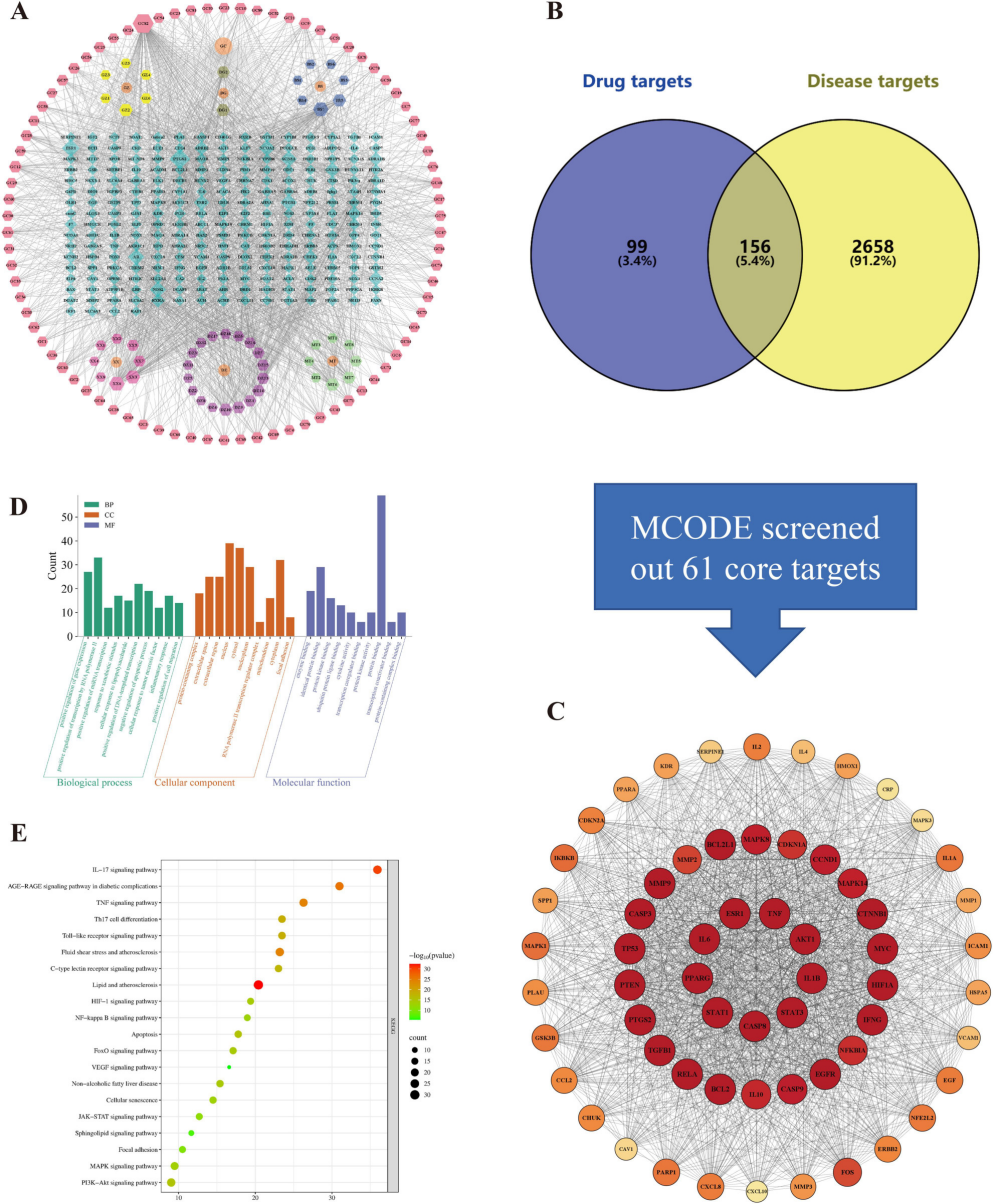
Network pharmacology analysis of DSG against CHD. **(A)** The “Drug-Active Ingredient-Target” (D-AI-T) network. **(B)** Venn diagram of overlapping targets between DSG and CHD. **(C)** Core protein-protein interaction (PPI) network. **(D)** Gene Ontology (GO) enrichment analysis. **(E)** KEGG pathway enrichment analysis.

Mapping the complex interactions between DSG's active compounds and biological targets required the construction of a “Drug-Active Ingredient-Target” (D-AI-T) network. Derived from public databases, this framework consists of 382 nodes and 1,962 edges (Figure 5A). Degree centrality evaluations highlighted Quercetin and Kaempferol as primary topological hubs. Crucially, the validity of these computational predictions was reinforced by our empirical UHPLC-Q-TOF-MS data (Table 2), where core network constituents like Quercetin, Kaempferol, Liquiritin, and Catechin were clearly identified within the actual DSG extract. This cross-validation anchors our theoretical model to a tangible chemical foundation.

A Venn diagram analysis revealed 156 shared targets between DSG active ingredients and established CHD genes (Figure 5B). To further isolate dense functional regions, these overlapping genes were processed through the STRING database and Cytoscape's MCODE plugin. The resulting high-scoring cluster (Score = 53.467) formed a core protein-protein interaction (PPI) network containing 61 nodes and 1,604 edges (Figure 5C). Hub proteins such as AKT1, BCL2, BCL2L1, and CASP3 ranked highest in MCODE scores, suggesting their pivotal role in the therapeutic mechanism (detailed in Supplementary Table S1).

Subsequent GO enrichment (Figure 5D) pointed towards biological processes including the “response to lipopolysaccharide,” “inflammatory response,” and “negative regulation of apoptotic process.” Molecular functions were primarily linked to protein and kinase binding, alongside cytokine activity. Furthermore, these targets were mostly situated in the nucleus, cytosol, and extracellular space. KEGG pathway analysis (Figure 5E) underscored the involvement of the IL-17, Lipid and atherosclerosis, PI3K-Akt, and MAPK signaling pathways. Together, these profiles imply that DSG exerts cardioprotection by modulating lipid metabolism, systemic hemodynamics, and inflammatory cascades.

### 3.4 DSG modulates gut microbiota composition

**Figure 6:**
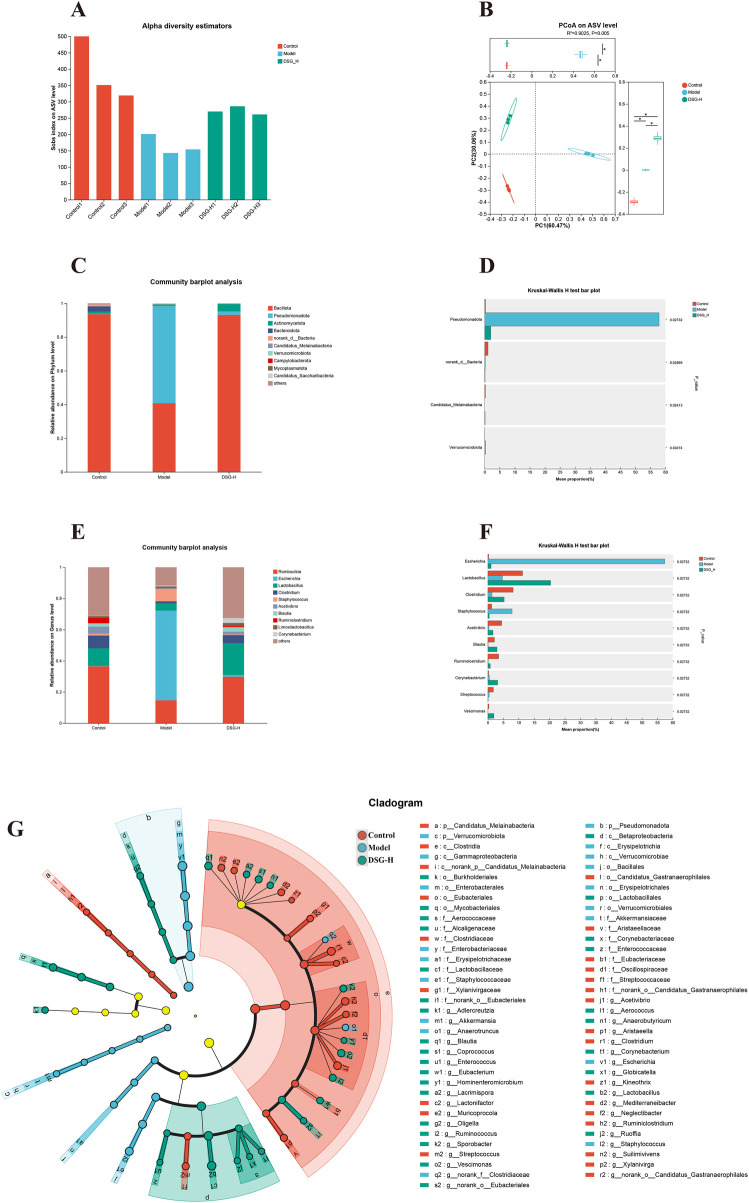
DSG modulates gut microbiota composition in CHD rats. **(A)** Alpha diversity evaluated by the Sobs index. **(B)** Principal Coordinate Analysis (PCoA) plot. **(C,D)** Relative abundance **(C)** and Kruskal–Wallis H test **(D)** of gut microbiota at the phylum level. **(E,F)** Relative abundance **(E)** and Kruskal–Wallis H test **(F)** at the genus level. **(G)** LEfSe cladogram identifying specific differential bacterial taxa (LDA > 2.0). For the 16S rRNA sequencing analysis, the sample size is *n* = 3 per group. Individual sample variations and data distribution are directly illustrated in the PCoA scatter plot **(B)** and the sample-wise bar plot **(A)**.

Since our network analysis highlighted lipid and inflammatory pathways, we sequenced the 16S rRNA gene to see if DSG changes the gut bacteria, which are key controllers of host metabolism and inflammation. The Sobs index (Figure 6A) showed that the Model rats lost a lot of microbial richness (*P* = 0.0183), but high-dose DSG helped recover it (*P* = 0.0049). The PCoA plot (Figure 6B) clearly separated the Model and Control groups. However, the DSG-H group shifted back toward the Control side, meaning the drug successfully repaired the damaged microbiota structure.Looking at the community bar plot (Figure 6C) and Kruskal–Wallis results (Figure 6D), Bacillota remained the top phylum for everyone. However, in the Model group, Pseudomonadota grew out of control and squeezed out Bacillota, a harmful expansion that was quickly stopped by DSG-H administration (overall Kruskal–Wallis *P* = 0.0273).

To pinpoint the specific bacterial taxa associated with these structural shifts, we initially examined the genus-level community composition (Figure 6E). To determine statistical significance and identify key structural biomarkers, we subsequently performed a Kruskal–Wallis test (Figure 6F) coupled with LEfSe analysis (Figure 6G). Using a strict screening threshold (LDA score > 2.0 and Kruskal–Wallis *P* < 0.05), we identified 35 differential genera (Table 3). Specifically, potentially pathogenic bacteria such as *Escherichia* (*P* = 0.0273)and *Staphylococcus* (*P* = 0.0273)were significantly enriched in the Model group, whereas the beneficial *Lactobacillus* was severely depleted (*P* = 0.0273). The LEfSe cladogram visually supported these taxa as defining markers for their respective groups. Furthermore, detailed pairwise comparisons (Table 3) revealed that out of the 35 altered genera, DSG-H treatment was associated with a significant reversal in the relative abundance of 14 core taxa that were dysregulated in the disease model. Together, these observations suggest that the cardioprotective profile of DSG is closely correlated with the modulation of these specific bacterial populations and the improvement of the intestinal microenvironment, providing supportive clues for its potential gut-heart-axis mechanisms.

**Table 3:** Effect of DSG-H on the relative abundance of key differential gut microbiota at the genus level.

No.	Bacteria (Genus)	Control (%)	Model (%)	DSG-H (%)	KW *P*-value	LDA Score	Enriched In	Model vs. Control	DSG-H vs. Model
1	*g__Escherichia*	0.26 ± 0.08	57.42 ± 7.88	1.05 ± 0.28	0.0273	5.45	Model	↑##	↓**
2	*g__Lactobacillus*	11.25 ± 1.01	4.71 ± 1.08	20.36 ± 2.22	0.0273	4.97	DSG-H	↓##	↑**
3	*g__Globicatella*	0.01 ± 0.02	0.00 ± 0.00	0.17 ± 0.01	0.0347	4.82	DSG-H	↓	↑**
4	*g__Staphylococcus*	1.25 ± 0.04	7.87 ± 2.04	0.44 ± 0.10	0.0273	4.58	Model	↑#	↓*
5	*g__Clostridium*	8.19 ± 0.67	1.34 ± 0.30	5.29 ± 0.88	0.0273	4.58	Control	↓###	↑**
6	*g__norank_f__Clostridiaceae*	0.01 ± 0.01	0.11 ± 0.03	0.00 ± 0.00	0.0340	4.53	Model	↑#	↓*
7	*g__Hominenteromicrobium*	0.02 ± 0.01	0.00 ± 0.00	0.13 ± 0.01	0.0340	4.51	DSG-H	↓	↑**
8	*g__Corynebacterium*	0.24 ± 0.12	0.51 ± 0.19	3.18 ± 0.13	0.0273	4.34	DSG-H	↑	↑***
9	*g__Acetivibrio*	4.48 ± 1.67	0.38 ± 0.20	1.67 ± 0.22	0.0273	4.33	Control	↓#	↑**
10	*g__Kineothrix*	0.07 ± 0.02	0.01 ± 0.02	0.00 ± 0.00	0.0336	4.31	Control	↓#	↓
11	*g__Blautia*	2.13 ± 0.22	0.08 ± 0.08	2.98 ± 0.32	0.0273	4.29	DSG-H	↓##	↑**
12	*g__Ruminiclostridium*	3.50 ± 1.01	0.11 ± 0.05	0.79 ± 0.06	0.0273	4.27	Control	↓#	↑***
13	*g__Oligella*	0.00 ± 0.00	0.04 ± 0.07	0.71 ± 0.07	0.0347	4.17	DSG-H	↑	↑***
14	*g__Vescimonas*	0.32 ± 0.06	0.02 ± 0.02	1.95 ± 0.51	0.0273	4.09	DSG-H	↓##	↑*
15	*g__Anaerobutyricum*	0.05 ± 0.04	0.00 ± 0.00	0.40 ± 0.04	0.0330	4.08	DSG-H	↓	↑**
16	*g__Enterococcus*	0.02 ± 0.04	0.00 ± 0.00	0.19 ± 0.04	0.0347	4.05	DSG-H	↓	↑*
17	*g__Coprococcus*	0.29 ± 0.09	0.00 ± 0.00	1.21 ± 0.11	0.0241	4.04	DSG-H	↓#	↑**
18	*g__Ruoffia*	0.00 ± 0.00	0.09 ± 0.10	0.34 ± 0.07	0.0340	4.03	DSG-H	↑	↑*
19	*g__Eubacterium*	0.36 ± 0.05	0.02 ± 0.03	0.49 ± 0.14	0.0379	4.02	DSG-H	↓##	↑*
20	*g__Anaerotruncus*	0.00 ± 0.00	0.51 ± 0.15	0.03 ± 0.03	0.0340	4.02	Model	↑#	↓*
21	*g__Akkermansia*	0.03 ± 0.05	0.34 ± 0.03	0.00 ± 0.00	0.0347	4.01	Model	↑##	↓**
22	*g__Xylanivirga*	0.39 ± 0.09	0.00 ± 0.00	0.02 ± 0.04	0.0347	3.98	Control	↓#	↑
23	*g__Lactonifactor*	0.17 ± 0.10	0.00 ± 0.00	0.05 ± 0.04	0.0340	3.97	Control	↓	↑
24	*g__Streptococcus*	1.79 ± 0.32	0.46 ± 0.27	0.10 ± 0.05	0.0273	3.97	Control	↓##	↓
25	*g__Mediterraneibacter*	0.76 ± 0.19	0.00 ± 0.00	0.01 ± 0.02	0.0347	3.96	Control	↓#	↑
26	*g__Aristaeella*	1.39 ± 0.25	0.07 ± 0.07	0.74 ± 0.18	0.0273	3.94	Control	↓##	↑*
27	*g__Ruminococcus*	0.22 ± 0.11	0.55 ± 0.06	1.20 ± 0.15	0.0273	3.92	DSG-H	↑#	↑**
28	*g__Muricoprocola*	0.35 ± 0.13	0.00 ± 0.00	0.02 ± 0.03	0.0347	3.89	Control	↓#	↑
29	*g__Lacrimispora*	0.04 ± 0.04	0.38 ± 0.03	0.54 ± 0.08	0.0273	3.88	DSG-H	↑###	↑
30	*g__norank_o__Candidatus_Gastranaerophilales*	0.27 ± 0.02	0.00 ± 0.00	0.15 ± 0.07	0.0241	3.81	Control	↓##	↑
31	*g__Suilimivivens*	1.06 ± 0.35	0.11 ± 0.09	0.00 ± 0.01	0.0265	3.8	Control	↓#	↓
32	*g__norank_o__Eubacteriales*	0.19 ± 0.03	0.17 ± 0.02	0.39 ± 0.09	0.0369	3.79	DSG-H	↓	↑*
33	*g__Adlercreutzia*	0.06 ± 0.06	0.01 ± 0.01	0.41 ± 0.16	0.0459	3.78	DSG-H	↓	↑*
34	*g__Sporobacter*	0.24 ± 0.05	0.73 ± 0.12	1.20 ± 0.47	0.0390	3.77	DSG-H	↑#	↑
35	*g__Neglectibacter*	0.37 ± 0.06	0.01 ± 0.02	0.24 ± 0.11	0.0379	3.76	Control	↓##	↑

Data are presented as mean ± SD (*n* = 3).

KW *P*-value represents the significance of the Kruskal–Wallis test among the three groups.

LDA Score was obtained from LEfSe analysis (LDA threshold >2.0).

# *P* < 0.05, ## *P* < 0.01, ### *P* < 0.001, the Model group vs. the Control group.

* *P* < 0.05, ** *P* < 0.01, *** *P* < 0.001, the DSG-H group vs. the Model group.

### 3.5 Serum metabolomics analysis

#### 3.5.1 Quality control and multivariate statistical analysis

**Figure 7:**
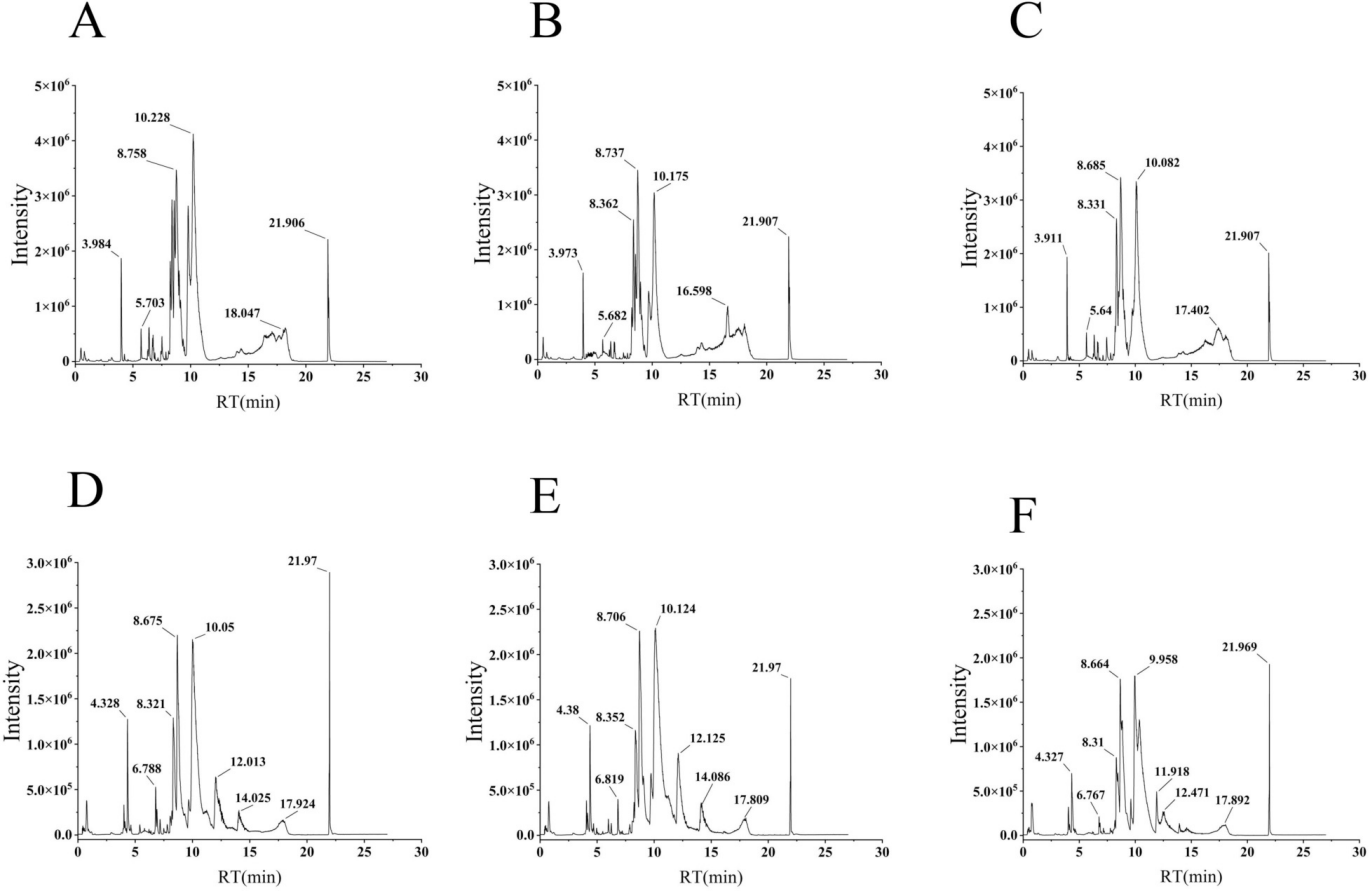
Serum metabolic profiles of the CHD rats. **(A–C)** Representative chromatograms in positive ionization mode for the Control **(A)**, Model **(B)**, and DSG-H **(C)** groups. **(D–F)** Representative chromatograms in negative ionization mode for the Control **(D)**, Model **(E)**, and DSG-H **(F)** groups.

**Figure 8:**
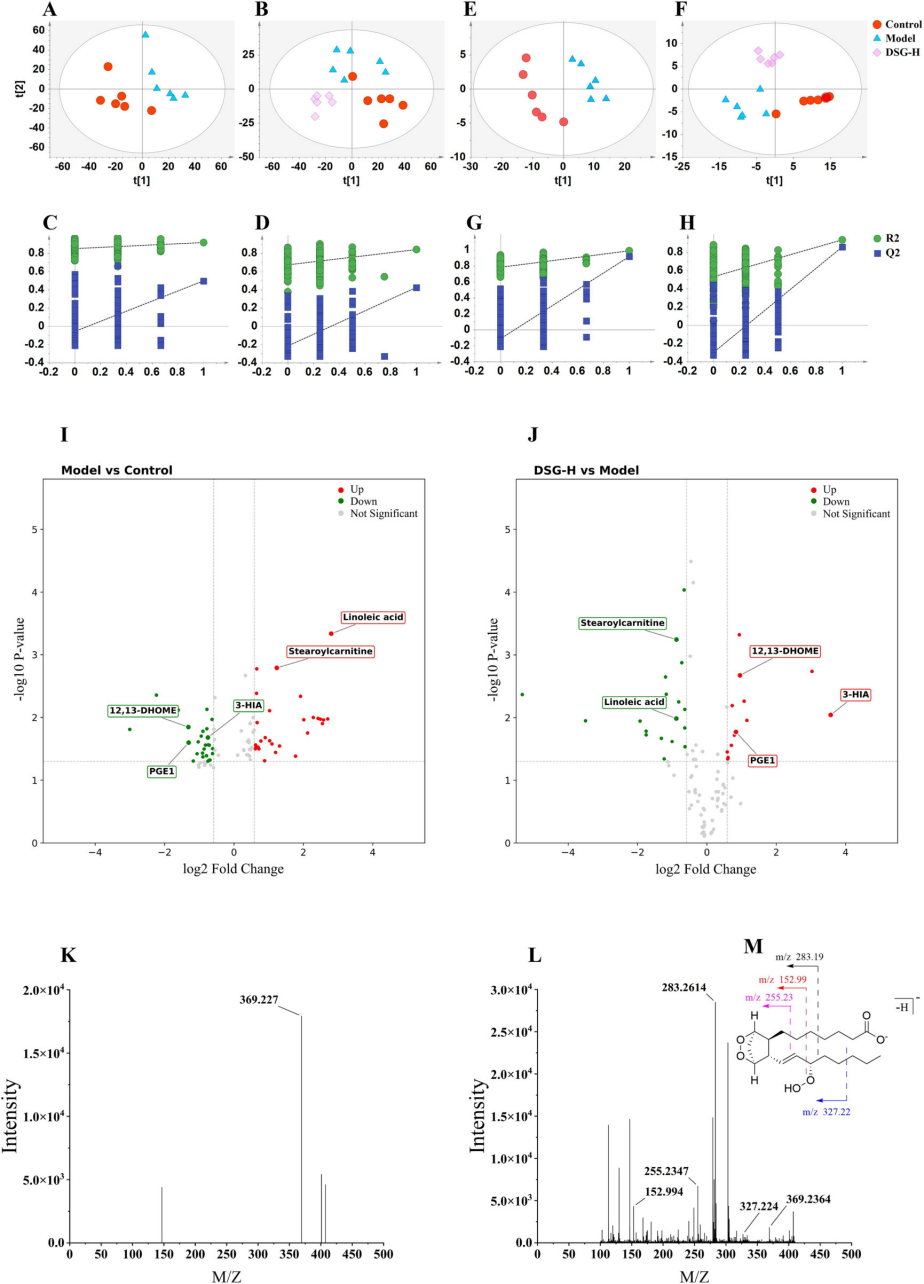
Multivariate statistical analysis and biomarker identification in serum metabolomics. **(A,B)** PLS-DA score plots in positive ionization mode for Control vs. Model **(A)** and Control, Model, and DSG-H groups **(B)**. **(C,D)** Permutation tests corresponding to **(A,B)**. **(E–H)** Corresponding PLS-DA score plots **(E,F)** and permutation tests **(G,H)** in negative ionization mode. **(I,J)** Volcano plots showing differentially expressed metabolites for Model vs. Control **(I)** and DSG-H vs. Model **(J) (K–M)** Representative MS **(K)** and MS/MS **(L)** spectra, alongside the proposed fragmentation pathway **(M)**, for the identification of prostaglandin G1. For the untargeted metabolomics analysis, the sample size is *n* = 6 per group. Individual sample variations and data distribution are directly illustrated in the multidimensional PLS-DA score plots.

System stability was first confirmed via quality control (QC) samples, with over 80% of ion features maintaining an RSD below 30%. Visual inspection of the total ion chromatograms (Figure 7) revealed distinct metabolic variations across the different experimental conditions. Subsequent PLS-DA score plots in positive ion mode (Figure 8A) completely segregated the Control and Model subjects, confirming severe metabolic disruption driven by the disease. The model's robustness was validated by high R2Y (0.962) and Q2 (0.446) scores (Supplementary Table S2), alongside rigorous permutation testing that ruled out overfitting (Figures 8C,D). Notably, the DSG-H group clustered separately from the Model group and shifted closer to the Controls (Figure 8B), indicating an overarching metabolic recovery. Parallel trends were observed under negative ionization (Figures 8E–H).

#### 3.5.2 Identification of potential biomarkers

To find the exact metabolites driving these changes, we combined VIP scores (VIP > 1) with Student's t-tests (*P* < 0.05) and fold-change limits (FC > 1.5 or <0.67). We used volcano plots to map this out: Figure 8I shows the metabolic mess caused by the CHD, and Figure 8J shows which metabolites reacted to the DSG-H. If a metabolite passed all three strict criteria, we tagged it as a potential biomarker.

Structural annotation was performed by matching accurate precursor masses (m/z) and MS/MS fragmentation patterns against the Human Metabolome Database (HMDB,http://www.hmdb.ca/). As a representative example, the identification of prostaglandin G1 (RT = 6.07 min, m/z 369.2270) is detailed in Figures 8K–M. The MS1 spectrum displayed a deprotonated molecular ion [M − H]⁻ at m/z 369.2270. In the MS/MS spectrum, characteristic fragment ions at m/z 327.2240 [(M − C_3_H_7_ − H)⁻] and 283.2614 (loss of butyric acid) were observed, along with fragments at m/z 255.2347 and 152.9940, confirming its structural identity. Through this systematic workflow, a total of 91 serum metabolites were identified (Supplementary Table S3). Crucially, comparative analysis revealed that 38 of these metabolites—which were significantly dysregulated in the Model group—exhibited a pronounced trend of reversal following DSG-H intervention. These “reversed metabolites” were identified as the key bioactive markers potentially mediating the therapeutic effects of DSG against CHD.

### 3.6 Joint pathway analysis

**Figure 9:**
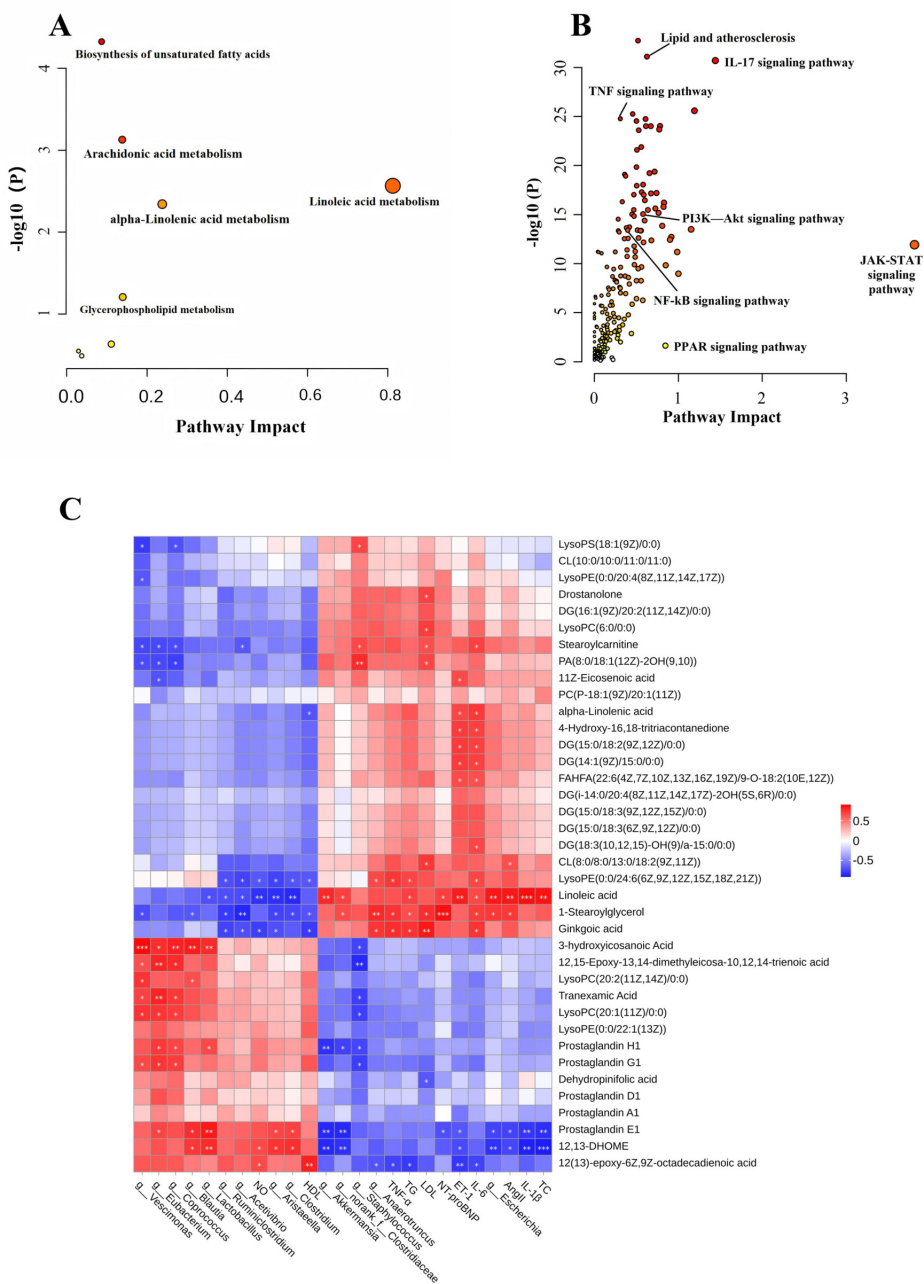
Joint pathway analysis and the correlation network of the microbiota-metabolite-phenotype axis. **(A,B)** Joint pathway enrichment analysis of the significantly reversed metabolites **(A)** and the core targets **(B)**. **(C)** Spearman correlation heatmap integrating the differential gut microbiota at the genus level, key serum metabolites, and clinical phenotypic markers. Red and blue indicate positive and negative correlations, respectively. Spearman correlation analysis was performed using 3 biologically paired samples per group (*n* = 3) across the microbiota, metabolome, and phenotype data. *P < 0.05, **P < 0.01, ***P < 0.001.

Integrating the 38 reversed metabolites with the core pharmacological targets yielded a comprehensive joint pathway map (Figures 9A,B). High enrichment significance was observed for disease-centric pathways like “Lipid and atherosclerosis,” alongside metabolic routes such as linoleic and arachidonic acid metabolism. While the “JAK-STAT signaling pathway” exhibited moderate enrichment significance, its unusually high Pathway Impact score suggests it acts as a critical topological hub for inflammatory signaling. The concurrent identification of the PPAR and PI3K-Akt pathways corroborated our earlier KEGG findings.

### 3.7 Integrated analysis of “microbiota-metabolite-phenotype” axis

To elucidate the “microbiota-metabolite-phenotype” axis, we constructed a Spearman correlation network (Figure 9C). For this correlation analysis, we focused on 14 core therapeutic targets derived from the 35 structural biomarkers (Table 3). These 14 genera were strictly selected based on a “double-significant reversal” criterion: they were significantly disrupted in the Model group (*P* < 0.05 vs. Control) and subsequently significantly reversed by DSG-H treatment (*P* < 0.05 vs. Model). Similarly, the differential metabolites included in this correlation analysis were the 38 key bioactive markers previously identified (as detailed in Section 3.5.2) that exhibited significant reversal following DSG-H intervention.

Using these strictly filtered variables, the resulting correlation matrix (|*ρ*| > 0.6, *P* < 0.05) positioned serum metabolites as a critical bridge between gut flora and clinical phenotypes. Specifically, beneficial bacterial genera like *Lactobacillus* and *Clostridium* correlated positively with cardioprotective metabolites such as 12,13-DHOME and PGE1 (exact *P* values ranging from 0.003 to 0.030), both of which were elevated following DSG-H therapy. Simultaneously, *Lactobacillus* exhibited a strong negative correlation with linoleic acid (*P* = 0.036), a pro-inflammatory lipid that accumulated in the Model rats. Furthermore, the beneficial metabolites (12,13-DHOME and PGE1) were inversely associated with vasoconstrictive factors like ET-1 and Ang II (all *P* < 0.05).

In contrast, the pathogenic genus *Escherichia*, which was significantly suppressed by DSG-H, exhibited a metabolic association profile highly consistent with pathological indices. It showed a significant positive correlation with the pro-inflammatory metabolite linoleic acid (*P* = 0.003), which, in turn, was positively correlated with inflammatory and lipid parameters, including IL-1β (*P* < 0.001), IL-6 (*P* = 0.042), TG (*P* = 0.030), and TC (*P* = 0.002). This co-variation pattern of “Potentially Opportunistic Taxa–Pro-inflammatory Metabolite–Pathological Index” suggests that DSG-H is associated with mitigated systemic inflammation and lipid dysregulation, potentially involving the reduction of *Escherichia* abundance and a concomitant decrease in the accumulation of pro-inflammatory lipids in the serum.

### 3.8 Molecular docking validation

**Figure 10:**
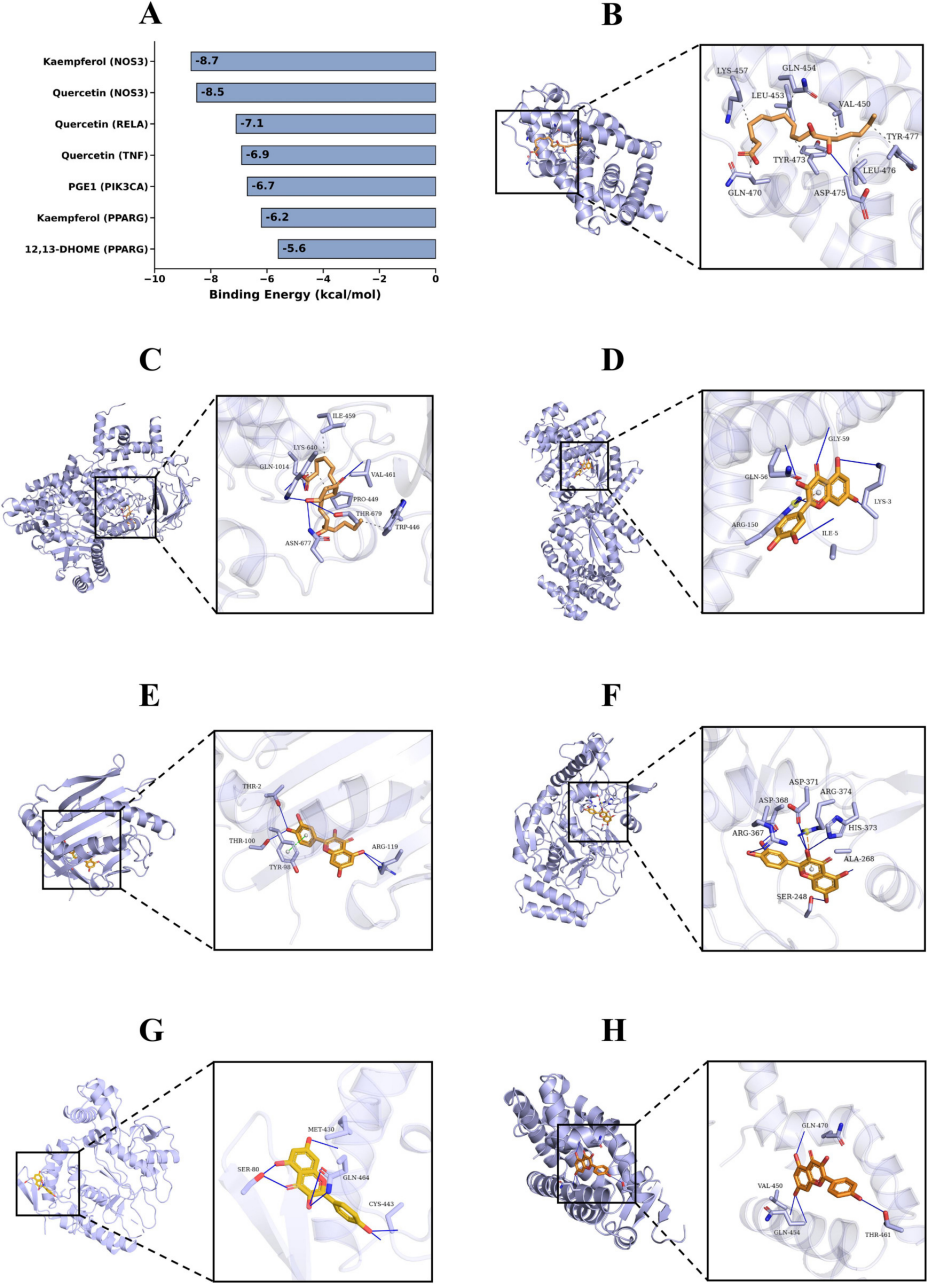
Molecular docking validation of key active components and lipid metabolites with their core targets. **(A)** The binding energy (kcal/mol) of the seven core docking combinations. A binding energy lower than −5.0 kcal/mol indicates a stable binding affinity. **(B–H)** 3D interaction models of the molecular docking. **(B)** 12,13-DHOME with PPARG. **(C)** PGE1 with PIK3CA. **(D)** Quercetin with RELA (NF-*κ*B p65). **(E)** Quercetin with TNF. **(F)** Quercetin with NOS3. **(G)** Kaempferol with NOS3. **(H)** Kaempferol with PPARG. The active components and lipid metabolites are presented in an orange stick model, while the target proteins are represented by translucent light blue cartoons. Solid blue lines indicate hydrogen bonds, and grey dashed lines represent hydrophobic interactions along with their corresponding interacting amino acid residues. Detailed molecular docking parameters are provided in Supplementary Table S4.

To explore the potential structural basis for our multi-omics correlational findings, molecular docking was employed to assess the theoretical binding capacities between the core ligands (Quercetin and Kaempferol, the primary topological hubs, along with the key reversed metabolites 12,13-DHOME and PGE1) and the key targets within the highly enriched pathways, including PIK3CA, NOS3, PPARG, TNF, and RELA. The docking results revealed robust binding affinities across the 7 assessed ligand-target pairs, all exhibiting binding energies well below the threshold of −5.0 kcal/mol, which indicates highly stable theoretical conformations (Figure 10). Visual inspection of the docking models revealed that these compounds could successfully occupy the active pockets of their respective proteins, primarily through the formation of hydrogen bonds and hydrophobic interactions with specific amino acid residues. While *in vivo* experimental validation remains necessary, these computational findings provide supporting theoretical clues for our systems biology predictions, suggesting that the constituents of DSG and their associated metabolites possess the structural potential to interact with these core signaling molecules.

## 4 Discussion

This study systematically evaluated the pharmacodynamic effects of DSG in a rat model of cold stress-exacerbated vasospastic CHD. The results showed that DSG significantly improved global pathological phenotypes. DSG attenuated ST-segment elevation and myocardial histopathological injury, improved dyslipidemia, and reduced systemic inflammatory mediators, including TNF-α and IL-6. These findings are generally consistent with previous reports on the anti-ischemic and angina-relieving effects of this formula (14, 38). Collectively, these data suggest that the cardioprotective effects of DSG are not limited to local myocardial changes but may involve coordinated systemic metabolic regulation. The gut-heart axis has emerged as an important topic in cardiovascular research, and gut microbiota are widely recognized as key modulators linking host metabolism and cardiovascular risk (39). However, whether DSG exerts anti-CHD effects through this axis has remained insufficiently characterized.

Notably, 16S rRNA sequencing suggested that DSG produced a bidirectional modulation of gut microbiota, consistent with the concept of bidirectional pharmacological regulation. This pattern differs from the single-direction approach of isolated prebiotic supplementation, such as stachyose (40). DSG reduced opportunistic/pathogenic genera, particularly *Escherichia* and *Staphylococcus*. Increased *Escherichia* abundance has been linked to plaque-related pathology in coronary disease (41), and some *Staphylococcus* strains or components may aggravate vascular inflammation and endothelial dysfunction (42, 43). Conversely, DSG enriched potentially beneficial genera, mainly *Lactobacillus* and *Clostridium*. Previous evidence associates *Lactobacillus* with a more favorable lipid profile and lower cardiovascular risk, and some strains may improve endothelial function in CHD (44, 45). Certain *Clostridium* species contribute to the production of short-chain fatty acids (SCFAs), including butyrate, as well as indole-3-propionic acid (IPA). IPA, a tryptophan-derived metabolite, has been associated with reduced platelet activation and thrombosis risk (15, 46). Together, these microbiota shifts provide a plausible microbial basis for the systemic cardioprotective profile of DSG.

Remodeling of the gut microbiota can drive changes in the host circulating metabolome, and microbiota–host co-metabolism is considered an important route through which TCM exerts protective effects on distant organs (47, 48). In this study, untargeted metabolomics showed that DSG significantly mitigated serum metabolic disturbances in CHD rats, mainly involving arachidonic acid and linoleic acid metabolism (Figure 9A). Based on differential-metabolite volcano plots in Figures 8I,J and multi-omics association analysis in Figure 9C, we observed that the Model group exhibited linoleic acid accumulation and reduced levels of the downstream protective metabolite 12,13-DHOME, whereas DSG intervention ameliorated this imbalance and increased 12,13-DHOME levels. This change was positively correlated with *Lactobacillus* abundance and was directionally consistent with previous reports (46). Because 12,13-DHOME is primarily generated by host CYP epoxygenases rather than direct bacterial production (49), DSG is more likely to restore this metabolic axis indirectly by improving the intestinal microenvironment and reducing inflammatory burden, including LPS-related inflammation (50). Meanwhile, the increase in 3-HIA suggested enhanced *β*-oxidation; together with the rise in 12,13-DHOME, this supports improved myocardial energy metabolism (51). At the level of arachidonic acid metabolism, DSG restored PGE1 levels, consistent with changes in *Clostridium* abundance. In light of the preceding findings, *Clostridium*-related metabolites, including SCFAs and IPA, may suppress systemic inflammation, reduce shunting of arachidonic acid toward pro-inflammatory pathways, and promote conversion toward the protective PGE1 branch (15, 46). Recovery of PGE1 showed negative association trends with ET-1, TG, and LDL-C, suggesting that DSG may improve the vascular microenvironment and lipid homeostasis (52). In addition, DSG-mediated suppression of *Escherichia* may further reduce pro-inflammatory lipid accumulation and support endothelial protection (53). Overall, by remodeling microbial taxa such as *Lactobacillus* and *Clostridium*, DSG may redirect metabolic flux toward beneficial lipid mediators, including 12,13-DHOME, 3-HIA, and PGE1, thereby promoting recovery of energy metabolism and microcirculatory function.

To further elucidate the signaling network underlying these metabolic phenotypes, we performed joint pathway enrichment analysis by integrating potential targets from network pharmacology with differential metabolites identified by metabolomics (Figure 9B). First, the Lipid and atherosclerosis pathway was among the more significantly enriched pathways in this study. This pathway integrates PPAR signaling with fatty acid metabolism and may be involved in DSG-mediated regulation of myocardial energy metabolism (54). Its enrichment was directionally consistent with remodeling of the linoleic acid–12,13-DHOME axis. 12,13-DHOME is a key oxylipin generated from linoleic acid via cytochrome P450 enzymes and can serve as an endogenous ligand-related signal for PPARs, particularly PPAR*γ* and PPAR*α* (49, 55). Recent evidence further emphasizes that such microbiota-modulated oxylipins act as critical signaling bridges that reprogram host cardiac lipid flux to confer stress resistance (56). Mechanistically, upon acting as a PPAR ligand, 12,13-DHOME promotes the heterodimerization of PPAR with the retinoid X receptor (RXR). This complex subsequently translocates to the nucleus and binds to specific PPAR response elements (PPREs) within the promoter regions of target metabolic genes (57, 58). Therefore, restoration of 12,13-DHOME after DSG intervention may help enhance PPAR-related pathway activity. Previous studies have shown that PPAR activation can upregulate the rate-limiting enzyme CPT-1, which facilitates long-chain fatty acid transport into mitochondria for β-oxidation, thereby rescuing the ischemic myocardium from energy starvation (59). Consistently, we observed recovery of the β-oxidation-related intermediate 3-hydroxyicosanoic acid (3-HIA), supporting the possibility that DSG promotes restoration of myocardial fatty acid oxidation flux and improves energy supply (60). Second, the PI3K-Akt pathway is an important signaling axis for endothelial cell survival and vasodilation (61). As described above, the vasodilatory factor PGE1 can bind specific G protein-coupled receptors, primarily the prostanoid EP (especially EP2 and EP4) and IP receptors, activate cAMP-PKA signaling, and crosstalk with PI3K-Akt signaling (62–64). Together with the observed increase in PGE1, these findings suggest that DSG may promote upstream activation of PI3K-Akt-related signaling. Activated Akt can increase phosphorylation of eNOS at Ser1177, enhance endogenous NO bioavailability, and thereby improve vasodilation and hemodynamics (65). Regulation of the PI3K-Akt/eNOS axis may therefore be one important mechanism by which DSG improves microcirculation and alleviates myocardial ischemia. In addition, joint analysis showed enrichment of inflammation-related pathways, including JAK-STAT and TNF/NF-*κ*B. This suggests that the anti-inflammatory effects of DSG may involve not only reductions in peripheral inflammatory mediators such as IL-6 but also attenuation of intracellular inflammatory signal amplification (66). This interpretation is consistent with the observed downward trends of TNF-α and IL-6 and supports the involvement of metabolism- and inflammation-related pathway regulation in DSG-associated cardioprotection.

Overall, DSG treatment in vasospastic CHD rats was associated with coordinated improvements in cardiac injury, dyslipidemia, endothelial-related dysfunction, and systemic inflammation. Integrated 16S rRNA profiling, metabolomics, and joint pathway analysis support a model in which DSG remodels gut microbiota composition and shifts host lipid metabolism toward protective mediators, including 12,13-DHOME, 3-HIA, and PGE1. To further explore the potential structural basis of these multi-omics findings, molecular docking was performed. The results suggested favorable theoretical binding affinities between the core hub ingredients, key rescued metabolites, and their respective targets within the PPAR, PI3K-Akt/eNOS, and inflammation-related signaling pathways. Together, this multidimensional approach offers a biologically plausible explanation for the cardioprotective profile of DSG from a gut-heart-axis perspective.

To the best of our knowledge, this is the first study to systematically investigate the protective mechanisms of DSG against cold stress-exacerbated vasospastic CHD from a holistic “microbiota-metabolite-phenotype” perspective, highlighting its systemic value beyond single-target pharmacology. However, it is important to acknowledge the specific characteristics and limitations of the current study. First, our animal model—combining a high-fat diet, cold stimulation, and posterior pituitary hormone (Pituitrin)—primarily simulates vasospastic CHD exacerbated by cold stress and lipid metabolic disorders. While the specific experimental procedure was adapted from a previous study (25), this tripartite design strictly captures the core pathophysiological triad of human vasospastic angina defined by the latest international cardiovascular guidelines: chronic endothelial dysfunction, environmental autonomic triggers, and acute vascular hyperreactivity (67). Although deliberately chosen to recapitulate the traditional clinical indications for DSG against “cold coagulation and blood stasis” (14, 38), this model is limited in its ability to observe advanced atheroma. Second, the current findings regarding the “microbiota-metabolite-phenotype” axis are primarily associative. Direct causal relationships have not yet been established through rigorous *in vivo* interventions, such as fecal microbiota transplantation (FMT). Third, regarding the mechanistic exploration, although we have introduced in silico molecular docking to computationally predict the binding affinities for the PI3K-Akt and NF-κB pathways, these mechanisms were not directly verified through protein-level biological experiments (e.g., Western blotting). Additionally, given the strictly limited sample size (*n* = 3 per group) for the 16S rRNA sequencing and specific biochemical assays: blood lipids and serum inflammatory cytokines, all multi-omics and pathway analysis results in this study should be viewed strictly as preliminary mechanistic explorations rather than definitive pharmacological evidence. Therefore, future studies are warranted to comprehensively validate these initial observations. Such investigations should include larger, standard pharmacological cohorts (*n* ≥ 6) and classic atherosclerotic models (e.g., ApoE-/- mice) to evaluate direct effects on plaque stability, alongside targeted biochemical interventions and microbiota-manipulation strategies to definitively confirm the causal links and exact molecular cascades.

## 5 Conclusions

DSG showed significant cardioprotective effects in a rat model of cold stress-exacerbated vasospastic CHD. DSG improved electrocardiographic and histopathological abnormalities, mitigated dyslipidemia, and attenuated systemic inflammation. Multi-omics integration further indicated that DSG was associated with gut microbiota remodeling and metabolic reprogramming, particularly in linoleic acid and arachidonic acid pathways, with restoration of levels of protective lipid mediators such as 12,13-DHOME, 3-HIA, and PGE1. Joint pathway analysis and in silico molecular docking suggested the potential involvement of PPAR-related metabolic regulation, PI3K-Akt/eNOS-mediated vascular protection, and inflammation-related signaling. Overall, these findings provide supportive evidence that DSG may exert cardioprotective effects through coordinated regulation of microbiota-related metabolism and inflammatory pathways. While the current associative multi-omics data and in silicocomputational predictions​ provide a preliminary suggestive structural rationale, key causal mechanisms should be definitively validated in future studies using targeted pathway intervention and microbiota-manipulation strategies.

## Data Availability

The original contributions presented in the study are included in the article/Supplementary Material, further inquiries can be directed to the corresponding author/s.

## Glossary

12,13-DHOME: 12,13-dihydroxy-9Z-octadecenoic acid
3-HIA: 3-Hydroxyicosanoic acid
ABCA1: ATP-binding cassette transporter A1
ABCG1: ATP-binding cassette transporter G1
Akt: Protein Kinase B
AKT1: AKT Serine/Threonine Kinase 1
Ang II: Angiotensin II
ASV: Amplicon Sequence Variant
BCL2: B-cell lymphoma 2
BCL2L1: BCL2 Like 1
BEH C18: Bridged Ethylene Hybrid C18
BP: Biological Process
cAMP: Cyclic Adenosine Monophosphate
CASP3: Caspase 3
CASP8: Caspase 8
CC: Cellular Component
CHD: Coronary Heart Disease
COX-2: Cyclooxygenase-2
CPT-1: Carnitine Palmitoyltransferase 1
CTNNβ1: Catenin Beta 1
DAB: 3,3′-Diaminobenzidine
DADA2: Divisive Amplicon Denoising Algorithm 2
D-AI-T: Drug—Active Ingredient—Target
DAPI: 4′,6-Diamidino-2-Phenylindole
DDA: Data-Dependent Acquisition
DL: Drug-Likeness
DSG: Danggui Sini Granules
EGFR: Epidermal Growth Factor Receptor
ELISA: Enzyme-Linked Immunosorbent Assay
eNOS: Endothelial nitric oxide synthase
EP receptors: Prostaglandin E_2_ Receptors
ET-1: Endothelin-1
FC: Fold Change
GO: Gene Ontology
HDL-C: High-Density Lipoprotein Cholesterol
HE: Hematoxylin and Eosin
HFD: High-Fat Diet
HIF-1: Hypoxia-Inducible Factor 1
HMDB: The Human Metabolome Database
IL-17: Interleukin-17
IL-1β: Interleukin-1 beta
IL-6: Interleukin-6
IPA: Indole-3-propionic acid
JAK-STAT: Janus Kinase—Signal Transducer and Activator of Transcription
KEGG: Kyoto Encyclopedia of Genes and Genomes
LC-MS: Liquid Chromatography-Mass Spectrometry
LDL-C: Low-Density Lipoprotein Cholesterol
LPS: Lipopolysaccharide
MAPK: Mitogen-Activated Protein Kinase
MF: Molecular Function
NCBI: National Center for Biotechnology Information
NF-κB: Nuclear Factor kappa B
NT-proBNP: N-terminal pro hormone of B-type natriuretic peptide
OB: Oral Bioavailability
OMIM: Online Mendelian Inheritance in Man
PBS: Phosphate Buffered Saline
PCoA: Principal Co-ordinates Analysis
PE reads: Paired-End Reads
PGE1: Prostaglandin E1
PI3K-Akt: Phosphatidylinositol 3-Kinase—Protein Kinase B
PKA: Protein Kinase A
PLS-DA: Partial Least Squares Discriminant Analysis
PPAR: Peroxisome Proliferator-Activated Receptor
PPARs: Peroxisome Proliferator-Activated Receptors
PPARα: Peroxisome Proliferator-Activated Receptor Alpha
PPARγ: Peroxisome Proliferator-Activated Receptor Gamma
PPI: Protein-Protein Interaction
QC: Quality Control
RSD: Relative Standard Deviation percentage
RT: Retention Time
SCFA: Short-Chain Fatty Acids
SRA: Sequence Read Archive
STAT: Signal Transducers and Activators of Transcription
STRING: Search Tool for the Retrieval of Interacting Genes
TC: Total Cholesterol
TCM: Traditional Chinese Medicine
TCMSP: Traditional Chinese Medicine Systems Pharmacology Database and Analysis Platform
TG: Triglyceride
TNF: Tumor Necrosis Factor
TNF-α: Tumor Necrosis Factor Alpha
TUNEL: Terminal deoxynucleotidyl transferase dUTP nick-end labeling
UHPLC: Ultra High Performance Liquid Chromatography
UPLC-MS: Ultra Performance Liquid Chromatography-Mass Spectrometry
VEGF: Vascular Endothelial Growth Factor
VIP: Variable Importance in the Projection.
