# Supplementary material for: Remodeling the gut-heart axis: Danggui Sini granule mitigates vasospastic coronary heart disease via microbiota-metabolite interactions
Source: Front Cardiovasc Med. 2026 May 22;13:1833846. doi: 10.3389/fcvm.2026.1833846 (PMC13236504; doi:10.3389/fcvm.2026.1833846)
Supplement: Supplementary file 1 [file Table1.docx]

**Table S1** The detailed node information of constructed networks

| No. | Gene names (primary) | Protein names |
| --- | --- | --- |
| 1 | MAPK1 | Mitogen-Activated Protein Kinase 1 |
| 2 | HMOX1 | Heme Oxygenase 1 |
| 3 | CXCL8 | Interleukin-8 |
| 4 | CXCL10 | C-X-C Motif Chemokine 10 |
| 5 | MMP3 | Stromelysin-1 |
| 6 | BCL2L1 | Bcl-2-Like Protein 1 |
| 7 | RELA | Transcription Factor P65 |
| 8 | AKT1 | Rac-Alpha Serine/Threonine-Protein Kinase |
| 9 | VCAM1 | Vascular Cell Adhesion Protein 1 |
| 10 | PLAU | Urokinase-Type Plasminogen Activator |
| 11 | TNF | Tumor Necrosis Factor |
| 12 | PTEN | Phosphatidylinositol 3,4,5-Trisphosphate 3-Phosphatase And Dual-Specificity Protein Phosphatase Pten |
| 13 | ICAM1 | Intercellular Adhesion Molecule 1 |
| 14 | PPARA | Peroxisome Proliferator-Activated Receptor Alpha |
| 15 | STAT3 | Signal Transducer And Activator Of Transcription 3 |
| 16 | CHUK | Inhibitor Of Nuclear Factor Kappa-B Kinase Subunit Alpha |
| 17 | IL1A | Interleukin-1 Alpha |
| 18 | MAPK3 | Mitogen-Activated Protein Kinase 3 |
| 19 | PARP1 | Poly |
| 20 | CRP | C-Reactive Protein |
| 21 | IL4 | Interleukin-4 |
| 22 | CASP8 | Caspase-8 |
| 23 | ERBB2 | Receptor Tyrosine-Protein Kinase Erbb-2 |
| 24 | IL2 | Interleukin-2 |
| 25 | PPARG | Peroxisome Proliferator-Activated Receptor Gamma |
| 26 | EGF | Pro-Epidermal Growth Factor |
| 27 | TP53 | Cellular Tumor Antigen P53 |
| 28 | SERPINE1 | Plasminogen Activator Inhibitor 1 |
| 29 | CCL2 | C-C Motif Chemokine 2 |
| 30 | CASP9 | Caspase-9 |
| 31 | TGFB1 | Transforming Growth Factor Beta-1 Proprotein |
| 32 | NFKBIA | Nf-Kappa-B Inhibitor Alpha |
| 33 | HSPA5 | Endoplasmic Reticulum Chaperone Bip |
| 34 | MMP1 | Interstitial Collagenase |
| 35 | ESR1 | Estrogen Receptor |
| 36 | IL6 | Interleukin-6 |
| 37 | HIF1A | Hypoxia-Inducible Factor 1-Alpha |
| 38 | KDR | Vascular Endothelial Growth Factor Receptor 2 |
| 39 | CASP3 | Caspase-3 |
| 40 | CTNNB1 | Catenin Beta-1 |
| 41 | IKBKB | Inhibitor Of Nuclear Factor Kappa-B Kinase Subunit Beta |
| 42 | GSK3B | Glycogen Synthase Kinase-3 Beta |
| 43 | PTGS2 | Prostaglandin G/H Synthase 2 |
| 44 | CAV1 | Caveolin-1 |
| 45 | CDKN1A | Cyclin-Dependent Kinase Inhibitor 1 |
| 46 | BCL2 | Apoptosis Regulator Bcl-2 |
| 47 | MYC | Myc Proto-Oncogene Protein |
| 48 | MAPK8 | Mitogen-Activated Protein Kinase 8 |
| 49 | IL10 | Interleukin-10 |
| 50 | MAPK14 | Mitogen-Activated Protein Kinase 14 |
| 51 | SPP1 | Osteopontin |
| 52 | CDKN2A | Tumor Suppressor Arf |
| 53 | CCND1 | G1/S-Specific Cyclin-D1 |
| 54 | FOS | Protein C-Fos |
| 55 | MMP9 | Matrix Metalloproteinase-9 |
| 56 | NFE2L2 | Nuclear Factor Erythroid 2-Related Factor 2 |
| 57 | EGFR | Epidermal Growth Factor Receptor |
| 58 | STAT1 | Signal Transducer And Activator Of Transcription 1-Alpha/Beta |
| 59 | IL1B | Interleukin-1 Beta |
| 60 | IFNG | Interferon Gamma |
| 61 | MMP2 | 72 Kda Type Iv Collagenase |
|  |  |  |
| No. | Node names | Detailed information |
| 1 | GC1 | Inermine |
| 2 | GC2 | Dfv |
| 3 | GC3 | Glycyrol |
| 4 | GC4 | Jaranol |
| 5 | GC5 | Medicarpin |
| 6 | GC6 | Isorhamnetin |
| 7 | GC7 | Sitosterol |
| 8 | GC8 | Lupiwighteone |
| 9 | GC9 | 7-Methoxy-2-Methyl Isoflavone |
| 10 | GC10 | Formononetin |
| 11 | GC11 | Calycosin |
| 12 | GC12 | Kaempferol |
| 13 | GC13 | (2S)-2-[4-Hydroxy-3-(3-Methylbut-2-Enyl)Phenyl]-8,8-Dimethyl-2,3-Dihydropyrano[2,3-F]Chromen-4-One |
| 14 | GC14 | Euchrenone |
| 15 | GC15 | Glyasperin B |
| 16 | GC16 | Glyasperin F |
| 17 | GC17 | Glyasperin C |
| 18 | GC18 | Isotrifoliol |
| 19 | GC19 | (E)-1-(2,4-Dihydroxyphenyl)-3-(2,2-Dimethylchromen-6-Yl)Prop-2-En-1-One |
| 20 | GC20 | Kanzonols W |
| 21 | GC21 | (2S)-6-(2,4-Dihydroxyphenyl)-2-(2-Hydroxypropan-2-Yl)-4-Methoxy-2,3-Dihydrofuro[3,2-G]Chromen-7-One |
| 22 | GC22 | Semilicoisoflavone B |
| 23 | GC23 | Glepidotin A |
| 24 | GC24 | Glepidotin B |
| 25 | GC25 | Phaseolinisoflavan |
| 26 | GC26 | Glypallichalcone |
| 27 | GC27 | 8-(6-Hydroxy-2-Benzofuranyl)-2,2-Dimethyl-5-Chromenol |
| 28 | GC28 | Licochalcone B |
| 29 | GC29 | Licochalcone G |
| 30 | GC30 | 3-(2,4-Dihydroxyphenyl)-8-(1,1-Dimethylprop-2-Enyl)-7-Hydroxy-5-Methoxy-Coumarin |
| 31 | GC31 | Licoricone |
| 32 | GC32 | Gancaonin A |
| 33 | GC33 | Gancaonin B |
| 34 | GC34 | 3-(3,4-Dihydroxyphenyl)-5,7-Dihydroxy-8-(3-Methylbut-2-Enyl)Chromone |
| 35 | GC35 | 5,7-Dihydroxy-3-(4-Methoxyphenyl)-8-(3-Methylbut-2-Enyl)Chromone |
| 36 | GC36 | 2-(3,4-Dihydroxyphenyl)-5,7-Dihydroxy-6-(3-Methylbut-2-Enyl)Chromone |
| 37 | GC37 | Glycyrin |
| 38 | GC38 | Licocoumarone |
| 39 | GC39 | Licoisoflavone |
| 40 | GC40 | Licoisoflavone B |
| 41 | GC41 | Licoisoflavanone |
| 42 | GC42 | Shinpterocarpin |
| 43 | GC43 | (E)-3-[3,4-Dihydroxy-5-(3-Methylbut-2-Enyl)Phenyl]-1-(2,4-Dihydroxyphenyl)Prop-2-En-1-One |
| 44 | GC44 | Liquiritin |
| 45 | GC45 | Licopyranocoumarin |
| 46 | GC46 | Glyzaglabrin |
| 47 | GC47 | Glabridin |
| 48 | GC48 | Glabrone |
| 49 | GC49 | 1,3-Dihydroxy-9-Methoxy-6-Benzofurano[3,2-C]Chromenone |
| 50 | GC50 | Eurycarpin A |
| 51 | GC51 | (-)-Medicocarpin |
| 52 | GC52 | Sigmoidin-B |
| 53 | GC53 | (2R)-7-Hydroxy-2-(4-Hydroxyphenyl)Chroman-4-One |
| 54 | GC54 | (2S)-7-Hydroxy-2-(4-Hydroxyphenyl)-8-(3-Methylbut-2-Enyl)Chroman-4-One |
| 55 | GC55 | Isoglycyrol |
| 56 | GC56 | Isolicoflavonol |
| 57 | GC57 | Hmo |
| 58 | GC58 | 1-Methoxyphaseollidin |
| 59 | GC59 | Quercetin Der. |
| 60 | GC60 | 3'-Hydroxy-4'-O-Methylglabridin |
| 61 | GC61 | Licochalcone A |
| 62 | GC62 | 3'-Methoxyglabridin |
| 63 | GC63 | 2-[(3R)-8,8-Dimethyl-3,4-Dihydro-2H-Pyrano[6,5-F]Chromen-3-Yl]-5-Methoxyphenol |
| 64 | GC64 | Inflacoumarin A |
| 65 | GC65 | Icos-5-Enoic Acid |
| 66 | GC66 | Kanzonol F |
| 67 | GC67 | 6-Prenylated Eriodictyol |
| 68 | GC68 | 7,2',4'-Trihydroxy－5-Methoxy-3－Arylcoumarin |
| 69 | GC69 | 7-Acetoxy-2-Methylisoflavone |
| 70 | GC70 | Gadelaidic Acid |
| 71 | GC71 | Vestitol |
| 72 | GC72 | Gancaonin G |
| 73 | GC73 | Gancaonin H |
| 74 | GC74 | Licoagrocarpin |
| 75 | GC75 | Glyasperins M |
| 76 | GC76 | Glycyrrhiza Flavonol A |
| 77 | GC77 | Licoagroisoflavone |
| 78 | GC78 | Odoratin |
| 79 | GC79 | Phaseol |
| 80 | GC80 | Xambioona |
| 81 | GC81 | Dehydroglyasperins C |
| 82 | GC82 | Quercetin |
| 83 | DG1 | Beta-Sitosterol |
| 84 | DG2 | Stigmasterol |
| 85 | GZ1 | (-)-Taxifolin |
| 86 | GZ2 | Beta-Sitosterol |
| 87 | GZ3 | Sitosterol |
| 88 | GZ4 | (+)-Catechin |
| 89 | GZ5 | Ent-Epicatechin |
| 90 | GZ6 | Taxifolin |
| 91 | BS1 | Paeoniflorgenone |
| 92 | BS2 | (3S,5R,8R,9R,10S,14S)-3,17-Dihydroxy-4,4,8,10,14-Pentamethyl-2,3,5,6,7,9-Hexahydro-1H-Cyclopenta[A]Phenanthrene-15,16-Dione |
| 93 | BS3 | Paeoniflorin |
| 94 | BS4 | Mairin |
| 95 | BS5 | Beta-Sitosterol |
| 96 | BS6 | Sitosterol |
| 97 | BS7 | Kaempferol |
| 98 | XX1 | 4,9-Dimethoxy-1-Vinyl-$B-Carboline |
| 99 | XX2 | Caribine |
| 100 | XX3 | Cryptopin |
| 101 | XX4 | Sesamin |
| 102 | XX5 | [(1S)-3-[(E)-But-2-Enyl]-2-Methyl-4-Oxo-1-Cyclopent-2-Enyl] (1R,3R)-3-[(E)-3-Methoxy-2-Methyl-3-Oxoprop-1-Enyl]-2,2-Dimethylcyclopropane-1-Carboxylate |
| 103 | XX6 | (3S)-7-Hydroxy-3-(2,3,4-Trimethoxyphenyl)Chroman-4-One |
| 104 | XX7 | Kaempferol |
| 105 | XX8 | Zinc05223929 |
| 106 | MT1 | Ariskanin A |
| 107 | MT2 | Poriferast-5-En-3Beta-Ol |
| 108 | MT3 | Hederagenin |
| 109 | MT4 | Beta-Sitosterol |
| 110 | MT5 | Stigmasterol |
| 111 | MT6 | Aristoloside_Qt |
| 112 | DZ1 | Stepharine |
| 113 | DZ2 | Zizyphus Saponin I_Qt |
| 114 | DZ3 | Coumestrol |
| 115 | DZ4 | Daechuine S7 |
| 116 | DZ5 | Jujubasaponin V_Qt |
| 117 | DZ6 | Mauritine D |
| 118 | DZ7 | Berberine |
| 119 | DZ8 | (S)-Coclaurine |
| 120 | DZ9 | Stigmasterol |
| 121 | DZ10 | Beta-Sitosterol |
| 122 | DZ11 | Ruvoside_Qt |
| 123 | DZ12 | (+)-Catechin |
| 124 | DZ13 | Stepholidine |
| 125 | DZ14 | Nuciferin |
| 126 | DZ15 | Fumarine |
| 127 | DZ16 | Beta-Carotene |
| 128 | DZ17 | (-)-Catechin |
| 129 | DZ18 | Quercetin |
