# Supplementary material for: Remodeling the gut-heart axis: Danggui Sini granule mitigates vasospastic coronary heart disease via microbiota-metabolite interactions
Source: Front Cardiovasc Med. 2026 May 22;13:1833846. doi: 10.3389/fcvm.2026.1833846 (PMC13236504; doi:10.3389/fcvm.2026.1833846)
Supplement: Supplementary file 2 [file Table2.docx]

**Table S2** The R2 Y and Q2 (cum) values

| Groups | R2 Y（cum） | Q2（cum） |
| --- | --- | --- |
| Figure 8A | 0.962 | 0.446 |
| Figure 8B | 0.817 | 0.410 |
| Figure 8C | 0.840 | 0.426 |
| Figure 8D | 0.825 | 0.423 |
| Figure 8E | 0.997 | 0.952 |
| Figure 8F | 0.921 | 0.822 |
| Figure 8G | 0.935 | 0.857 |
| Figure 8H | 0.904 | 0.815 |
