# Supplementary material for: Remodeling the gut-heart axis: Danggui Sini granule mitigates vasospastic coronary heart disease via microbiota-metabolite interactions
Source: Front Cardiovasc Med. 2026 May 22;13:1833846. doi: 10.3389/fcvm.2026.1833846 (PMC13236504; doi:10.3389/fcvm.2026.1833846)
Supplement: Supplementary file 3 [file Table3.docx]

**Table S3** Identification of potential metabolic biomarkers in rat serum and their regulatory trends by DSG-H

| No. | Metabolite | Retention time (min) | Chemical Formula | Detected m/z | Mass Error  (ppm) | Adducts | HMDB ID | Model vs Control | DSG-H vs Model |
| --- | --- | --- | --- | --- | --- | --- | --- | --- | --- |
| 1 | Glycoursodeoxycholic acid | 6.14 | C26H43NO5 | 472.3041 | 1.65 | [M+Na]+ | HMDB0000708 | ↓# | ↑ |
| 2 | Stearoylcarnitine | 9.01 | C25H49NO4 | 428.3742 | 1.70 | [M+H]+ | HMDB0000848 | ↑# | ↓* |
| 3 | alpha-Linolenic acid | 15.80 | C18H30O2 | 557.4571 | 1.26 | [2M+H]+ | HMDB0001388 | ↑## | ↓* |
| 4 | Prostaglandin E1 | 7.47 | C20H34O5 | 337.2367 | -1.72 | [M+H-H2O]+ | HMDB0001442 | ↓# | ↑* |
| 5 | Eicosapentaenoic acid | 8.78 | C20H30O2 | 303.2326 | 2.45 | [M+H]+ | HMDB0001999 | ↓# | ↑ |
| 6 | Tryptophol | 10.12 | C10H11NO | 184.0738 | 3.23 | [M+Na]+ | HMDB0003447 | ↑# | ↓ |
| 7 | 16(R)-HETE | 8.59 | C20H32O3 | 343.2248 | 1.29 | [M+Na]+ | HMDB0004680 | ↓# | ↓ |
| 8 | PC(20:0/14:1(9Z)) | 14.24 | C42H82NO8P | 760.5848 | -0.41 | [M+H]+ | HMDB0008263 | ↑# | ↓ |
| 9 | PC(22:0/24:0) | 0.41 | C54H108NO8P | 912.7814 | 3.65 | [M+H-H2O]+ | HMDB0008552 | ↑# | ↑* |
| 10 | PC(22:6(4Z,7Z,10Z,13Z,16Z,19Z)/20:4(5Z,8Z,11Z,14Z)) | 18.38 | C50H80NO8P | 854.5716 | 2.49 | [M+H]+ | HMDB0008739 | ↑# | ↑ |
| 11 | LysoPC(16:0/0:0) | 17.89 | C24H50NO7P | 478.3304 | 2.36 | [M+H-H2O]+ | HMDB0010382 | ↓## | ↓** |
| 12 | LysoPC(22:6(4Z,7Z,10Z,13Z,16Z,19Z)/0:0) | 8.28 | C30H50NO7P | 590.3222 | 0.78 | [M+Na]+ | HMDB0010404 | ↑# | ↓ |
| 13 | Isoleucylproline | 0.88 | C11H20N2O3 | 229.1548 | 0.68 | [M+H]+ | HMDB0011174 | ↑# | ↓ |
| 14 | LysoPE(0:0/18:1(11Z)) | 8.54 | C23H46NO7P | 462.2974 | -0.96 | [M+H-H2O]+ | HMDB0011475 | ↓# | ↑ |
| 15 | LysoPE(0:0/20:1(11Z)) | 8.47 | C25H50NO7P | 530.3233 | 3.07 | [M+Na]+ | HMDB0011482 | ↑## | ↓ |
| 16 | SM(d18:0/18:1(11Z)) | 18.08 | C41H83N2O6P | 753.5864 | -2.29 | [M+Na]+ | HMDB0012088 | ↑# | ↓ |
| 17 | SM(d18:0/24:0) | 18.59 | C47H97N2O6P | 834.7353 | -8.56 | [M+NH4]+ | HMDB0012094 | ↑# | ↓ |
| 18 | Myristoylglycine | 8.50 | C16H31NO3 | 571.4705 | 4.27 | [2M+H]+ | HMDB0013250 | ↑# | ↓ |
| 19 | Tranexamic Acid | 0.75 | C8H15NO2 | 158.1182 | 3.97 | [M+H]+ | HMDB0014447 | ↓## | ↑*** |
| 20 | Drostanolone | 12.20 | C20H32O2 | 305.2478 | 1.05 | [M+H]+ | HMDB0014996 | ↑## | ↓* |
| 21 | LysoPC(6:0/0:0) | 11.81 | C14H30NO7P | 373.2121 | 6.40 | [M+NH4]+ | HMDB0029207 | ↑# | ↓* |
| 22 | Ginkgoic acid | 11.83 | C22H34O3 | 329.2481 | 1.59 | [M+H-H2O]+ | HMDB0033897 | ↑## | ↓* |
| 23 | TG(14:1(9Z)/18:4(6Z,9Z,12Z,15Z)/18:2(9Z,12Z)) | 8.78 | C53H88O6 | 838.6925 | 0.73 | [M+NH4]+ | HMDB0048284 | ↓# | ↑ |
| 24 | DG(14:1n5/0:0/20:5n3) | 13.95 | C37H60O5 | 567.4360 | -8.20 | [M+H-H2O]+ | HMDB0056153 | ↑## | ↓ |
| 25 | 12(13)-epoxy-6Z,9Z-octadecadienoic acid | 8.29 | C18H30O3 | 277.2159 | -1.14 | [M+H-H2O]+ | HMDB0062288 | ↓# | ↑* |
| 26 | (10E,12Z)-9-HODE | 7.47 | C18H32O3 | 279.2315 | -1.17 | [M+H-H2O]+ | HMDB0062652 | ↓# | ↑ |
| 27 | TG(i-24:0/22:0/8:0) | 4.26 | C57H110O6 | 891.8462 | 9.70 | [M+H]+ | HMDB0070800 | ↓# | ↑ |
| 28 | CL(8:0/8:0/13:0/18:2(9Z,11Z)) | 8.29 | C56H104O17P2 | 1133.6554 | -7.84 | [M+Na]+ | HMDB0116977 | ↑## | ↓* |
| 29 | CL(10:0/10:0/11:0/11:0) | 8.30 | C51H98O17P2 | 1045.6349 | -0.28 | [M+H]+ | HMDB0124594 | ↑# | ↓** |
| 30 | LysoPS(18:1(9Z)/0:0) | 8.29 | C24H46NO9P | 524.2955 | -5.37 | [M+H]+ | HMDB0240603 | ↑# | ↓*** |
| 31 | SM(d18:1/17:0) | 16.56 | C40H81N2O6P | 717.5889 | -2.19 | [M+H]+ | HMDB0240609 | ↑# | ↓ |
| 32 | 3-MethylHexadecanoylcarnitine | 8.56 | C24H47NO4 | 414.3589 | 2.70 | [M+H]+ | HMDB0240862 | ↑# | ↓ |
| 33 | 1-Stearoylglycerol | 10.09 | C21H42O4 | 341.3023 | -7.53 | [M+H-H2O]+ | HMDB0244009 | ↑## | ↓** |
| 34 | Bax inhibitor peptide V5 | 8.28 | C27H50N6O6S | 569.3429 | -8.70 | [M+H-H2O]+ | HMDB0248888 | ↑## | ↓ |
| 35 | S-Petasin | 18.00 | C19H26O3S | 335.1680 | 1.45 | [M+H]+ | HMDB0257423 | ↓## | ↓*** |
| 36 | PA(8:0/18:1(12Z)-2OH(9,10)) | 5.39 | C29H55O10P | 595.3634 | 4.82 | [M+H]+ | HMDB0266641 | ↑# | ↓** |
| 37 | PGP(20:4(5Z,8Z,10E,14Z)-OH(12S)/a-25:0) | 8.69 | C51H94O14P2 | 1010.6548 | 9.21 | [M+NH4]+ | HMDB0274700 | ↑# | ↓ |
| 38 | PS(20:1(11Z)/20:3(6,8,11)-OH(5)) | 8.78 | C46H82NO11P | 838.5575 | -2.09 | [M+H-H2O]+ | HMDB0282247 | ↓# | ↑ |
| 39 | PC(18:1(11Z)/22:6(4Z,7Z,11E,13Z,15E,19Z)-2OH(10S,17)) | 8.91 | C48H82NO10P | 886.5553 | -1.77 | [M+Na]+ | HMDB0286395 | ↓# | ↑ |
| 40 | PC(20:0/20:5(5Z,8Z,11Z,14Z,16E)-OH(18R)) | 18.59 | C48H86NO9P | 834.5994 | -1.57 | [M+H-H2O]+ | HMDB0286994 | ↑# | ↓ |
| 41 | PC(22:1(13Z)/LTE4) | 8.31 | C53H95N2O11PS | 1021.6279 | -0.75 | [M+Na]+ | HMDB0287901 | ↑# | ↓ |
| 42 | Cer(d16:1/5-iso PGF2VI) | 17.98 | C34H61NO6 | 597.4811 | -4.46 | [M+NH4]+ | HMDB0289859 | ↑## | ↓ |
| 43 | DG(PGD2/a-25:0/0:0) | 10.16 | C48H86O8 | 808.6735 | 9.33 | [M+NH4]+ | HMDB0298155 | ↑# | ↑ |
| 44 | L-threo-sphinganine | 8.78 | C18H40NO2+ | 303.3121 | -3.67 | [M+H]+ | HMDB0304406 | ↓# | ↑ |
| 45 | Linoleic acid | 19.55 | C18H32O2 | 559.4714 | -3.14 | [2M-H]- | HMDB0000673 | ↑# | ↓* |
| 46 | Prostaglandin H2 | 8.24 | C20H32O5 | 333.2048 | -6.60 | [M-H2O-H]- | HMDB0001381 | ↓# | ↑ |
| 47 | Docosahexaenoic acid | 8.23 | C22H32O2 | 327.2324 | -1.73 | [M-H]- | HMDB0002183 | ↑# | ↑** |
| 48 | 11Z-Eicosenoic acid | 13.54 | C20H38O2 | 619.5727 | 9.12 | [2M-H]- | HMDB0002231 | ↑# | ↓* |
| 49 | Prostaglandin A1 | 8.24 | C20H32O4 | 317.2124 | 0.68 | [M-H2O-H]- | HMDB0002656 | ↓# | ↑* |
| 50 | 12,13-DHOME | 8.54 | C18H34O4 | 295.2258 | -6.63 | [M-H2O-H]- | HMDB0004705 | ↓# | ↑* |
| 51 | Prostaglandin D1 | 6.13 | C20H34O5 | 353.2323 | -2.84 | [M-H]- | HMDB0005102 | ↓# | ↑* |
| 52 | 12-HETE | 8.88 | C20H32O3 | 319.2287 | 2.60 | [M-H]- | HMDB0006111 | ↓# | ↑ |
| 53 | DG(14:1(9Z)/15:0/0:0) | 18.28 | C32H60O5 | 505.4247 | -2.99 | [M-H2O-H]- | HMDB0007039 | ↑## | ↓* |
| 54 | DG(15:0/18:2(9Z,12Z)/0:0) | 18.19 | C36H66O5 | 577.4817 | -3.61 | [M-H]- | HMDB0007074 | ↑## | ↓** |
| 55 | DG(15:0/18:3(6Z,9Z,12Z)/0:0) | 18.37 | C36H64O5 | 557.4558 | -3.05 | [M-H2O-H]- | HMDB0007075 | ↑# | ↓** |
| 56 | DG(15:0/18:3(9Z,12Z,15Z)/0:0) | 16.14 | C36H64O5 | 575.4660 | -3.63 | [M-H]- | HMDB0007076 | ↑## | ↓** |
| 57 | DG(16:1(9Z)/20:2(11Z,14Z)/0:0) | 20.37 | C39H70O5 | 599.4998 | -7.56 | [M-H2O-H]- | HMDB0007138 | ↑# | ↓* |
| 58 | LysoPC(20:1(11Z)/0:0) | 10.11 | C28H56NO7P | 594.3763 | -2.49 | [M+FA-H]- | HMDB0010391 | ↓## | ↑** |
| 59 | LysoPC(20:2(11Z,14Z)/0:0) | 9.35 | C28H54NO7P | 592.3574 | -8.44 | [M+FA-H]- | HMDB0010392 | ↓# | ↑* |
| 60 | LysoPC(24:1(15Z)/0:0) | 9.99 | C32H64NO7P | 640.4070 | -7.39 | [M+Cl]- | HMDB0010406 | ↑## | ↓ |
| 61 | PC(P-18:1(9Z)/20:1(11Z)) | 0.42 | C46H88NO7P | 832.6029 | 4.54 | [M+Cl]- | HMDB0011315 | ↑# | ↓* |
| 62 | LysoPE(0:0/18:1(11Z)) | 8.47 | C23H46NO7P | 460.2831 | -0.48 | [M-H2O-H]- | HMDB0011475 | ↓# | ↑ |
| 63 | LysoPE(0:0/20:4(8Z,11Z,14Z,17Z)) | 8.25 | C25H44NO7P | 500.2784 | 0.19 | [M-H]- | HMDB0011488 | ↑# | ↓** |
| 64 | LysoPE(0:0/22:1(13Z)) | 10.11 | C27H54NO7P | 534.3552 | -2.40 | [M-H]- | HMDB0011491 | ↓# | ↑** |
| 65 | LysoPE(0:0/24:6(6Z,9Z,12Z,15Z,18Z,21Z)) | 8.24 | C29H48NO7P | 552.3118 | 4.13 | [M-H]- | HMDB0011499 | ↑# | ↓* |
| 66 | MG(0:0/22:0/0:0) | 6.93 | C25H50O4 | 459.3707 | 3.73 | [M+FA-H]- | HMDB0011551 | ↓## | ↑ |
| 67 | Prostaglandin G1 | 6.07 | C20H34O6 | 369.2271 | -3.19 | [M-H]- | HMDB0013039 | ↓# | ↑* |
| 68 | Prostaglandin H1 | 6.64 | C20H34O5 | 353.2327 | -1.77 | [M-H]- | HMDB0013041 | ↓# | ↑** |
| 69 | S-(9-hydroxy-PGA1)-glutathione | 9.14 | C30H51N3O10S | 626.3104 | -2.01 | [M-H2O-H]- | HMDB0013059 | ↑# | ↓ |
| 70 | 6,8-Tricosanedione | 8.89 | C23H44O2 | 387.3046 | 3.00 | [M+Cl]- | HMDB0035564 | ↓# | ↑ |
| 71 | Dehydropinifolic acid | 7.91 | C20H30O4 | 333.2058 | -3.98 | [M-H]- | HMDB0036828 | ↓# | ↑* |
| 72 | (E)-11-Hexadecenoic acid | 12.00 | C16H30O2 | 253.2172 | -0.49 | [M-H]- | HMDB0037647 | ↓# | ↑ |
| 73 | 4-Hydroxy-16,18-tritriacontanedione | 18.14 | C33H64O3 | 553.4813 | -4.79 | [M+FA-H]- | HMDB0039536 | ↑# | ↓* |
| 74 | 5-(14-Heneicosenyl)-1,3-benzenediol | 14.28 | C27H46O2 | 447.3465 | -3.71 | [M+FA-H]- | HMDB0039869 | ↑## | ↓ |
| 75 | 12-Oxo-c-LTB3 | 9.96 | C30H47N3O10S | 640.2919 | 1.47 | [M-H]- | HMDB0060154 | ↑## | ↓ |
| 76 | 3-hydroxyicosanoic Acid | 10.11 | C20H40O3 | 309.2781 | -5.50 | [M-H2O-H]- | HMDB0062566 | ↓# | ↑** |
| 77 | 14-oxo-DoHE(1-) | 8.92 | C22H30O3 | 387.2153 | -7.10 | [M+FA-H]- | HMDB0062685 | ↓# | ↑ |
| 78 | 12,15-Epoxy-13,14-dimethyleicosa-10,12,14-trienoic acid | 9.37 | C22H36O3 | 347.2585 | -1.87 | [M-H]- | HMDB0112069 | ↓# | ↑*** |
| 79 | FAHFA(22:6(4Z,7Z,10Z,13Z,16Z,19Z)/9-O-18:2(10E,12Z)) | 13.52 | C40H62O4 | 641.4378 | 5.97 | [M+Cl]- | HMDB0112169 | ↑# | ↓* |
| 80 | PS(22:4(7Z,10Z,13Z,16Z)/20:2(11Z,14Z)) | 9.04 | C48H82NO10P | 908.5626 | -3.75 | [M+FA-H]- | HMDB0112802 | ↓# | ↑ |
| 81 | PA(15:0/18:3(9Z,12Z,15Z)) | 8.68 | C36H65O8P | 1357.8944 | 9.77 | [2M+FA-H]- | HMDB0114817 | ↓## | ↑ |
| 82 | PA(18:1(11Z)/18:0) | 17.94 | C39H75O8P | 701.5134 | 1.01 | [M-H]- | HMDB0114901 | ↓## | ↓*** |
| 83 | PA(18:1(9Z)/14:1(9Z)) | 8.68 | C35H65O8P | 1333.8893 | 6.00 | [2M+FA-H]- | HMDB0114922 | ↓# | ↑ |
| 84 | trans-1,4-Bis[[1-cyclohexyl-3-(4-dimethylamino phenyl)ureido]methyl]cyclohexane | 11.80 | C38H58N6O2 | 629.4529 | -3.14 | [M-H]- | HMDB0255813 | ↑# | ↓ |
| 85 | PS(P-20:0/22:6(4Z,7Z,10Z,13Z,16Z,19Z)) | 8.91 | C48H82NO9P | 882.5480 | 6.98 | [M+Cl]- | HMDB0256890 | ↓# | ↑ |
| 86 | PA(8:0/18:1(12Z)-2OH(9,10)) | 8.23 | C29H55O10P | 629.3201 | -4.31 | [M+Cl]- | HMDB0266641 | ↑# | ↓ |
| 87 | PA(8:0/22:6(5Z,8E,10Z,13Z,15E,19Z)-2OH(7S, 17S)) | 7.19 | C33H53O10P | 621.3142 | -8.71 | [M-H2O-H]- | HMDB0266655 | ↓# | ↑ |
| 88 | DG(10:0/18:1(12Z)-2OH(9,10)/0:0) | 8.45 | C31H58O7 | 1083.8214 | -7.21 | [2M-H]- | HMDB0294513 | ↑# | ↓ |
| 89 | DG(18:3(10,12,15)-OH(9)/a-15:0/0:0) | 15.43 | C36H64O6 | 573.4505 | -3.34 | [M-H2O-H]- | HMDB0297656 | ↑# | ↓* |
| 90 | DG(i-14:0/20:4(8Z,11Z,14Z,17Z)-2OH(5S,6R)/0:0) | 13.47 | C37H64O7 | 619.4562 | -2.84 | [M-H]- | HMDB0298873 | ↑# | ↓* |
| 91 | Hexadecadienoic acid | 18.05 | C16H28O2 | 503.4099 | -1.45 | [2M-H]- | HMDB0302694 | ↑## | ↓ |

# *p* < 0.05, ## *p* < 0.01, ### *p* < 0.001, the Model group versus the Control group.

* *p* < 0.05, ** *p* < 0.01, *** *p* < 0.001, the DSG-H group versus the Model group.
