# Supplementary material for: Remodeling the gut-heart axis: Danggui Sini granule mitigates vasospastic coronary heart disease via microbiota-metabolite interactions
Source: Front Cardiovasc Med. 2026 May 22;13:1833846. doi: 10.3389/fcvm.2026.1833846 (PMC13236504; doi:10.3389/fcvm.2026.1833846)
Supplement: Supplementary file 4 [file Table4.docx]

**Table S4** Detailed molecular docking parameters of key active components and lipid metabolites with their core targets.

| Components | Targets | Binding Energy  (kcal/mol) | No. of H-bonds | Interacting Residues |
| --- | --- | --- | --- | --- |
| Kaempferol | NOS3 | -8.7 | 8 | SER:80, MET:430, CYS:443, GLN:464 |
| Quercetin | NOS3 | -8.5 | 7 | SER:248, ALA:268, ARG:367, ASP:368, ASP:371, HIS:373, ARG:374 |
| Quercetin | RELA | -7.1 | 6 | LYS:3, ILE:5, GLN:56, GLY:59, ARG:150 |
| Quercetin | TNF | -6.9 | 4 | THR:2, THR:100, ARG:119, TYR:98 |
| PGE1 | PIK3CA | -6.7 | 7 | VAL:461, LYS:640, ASN:677, THR:679, GLY:1009, GLN:1014 |
| Kaempferol | PPARG | -6.2 | 4 | VAL:450, GLN:454, THR:461, GLN:470 |
| 12,13-DHOME | PPARG | -5.6 | 1 | ASP:475 |
